# Comparative analysis of the microbiomes of strawberry wild species *Fragaria nilgerrensis* and cultivated variety Akihime using amplicon-based next-generation sequencing

**DOI:** 10.3389/fmicb.2024.1377782

**Published:** 2024-05-30

**Authors:** Zongneng Wang, Qingzhong Dai, Daifa Su, Zhenrong Zhang, Yunxia Tian, Jiangyun Tong, Shanyan Chen, Congwen Yan, Junyu Yang, Xiaolong Cui

**Affiliations:** ^1^Yunnan Institute of Microbiology, School of Life Sciences, Yunnan University, Kunming, China; ^2^State Key Laboratory for Conservation and Utilization of Bio-Resources in Yunnan, Yunnan University, Kunming, China; ^3^Kunming Academy of Agricultural Science, Kunming, China; ^4^Yunnan International Joint Laboratory of Virology and Immunology, Kunming, China

**Keywords:** *Fragaria nilgerrensis*, cultivated strawberry, fungi, bacteria, microbial diversity of strawberry

## Abstract

*Fragaria nilgerrensis* is a wild strawberry species widely distributed in southwest China and has strong ecological adaptability. Akihime (*F. × ananassa* Duch. cv. Akihime) is one of the main cultivated strawberry varieties in China and is prone to infection with a variety of diseases. In this study, high-throughput sequencing was used to analyze and compare the soil and root microbiomes of *F. nilgerrensis* and *Akihime.* Results indicate that the wild species *F. nilgerrensis* showed higher microbial diversity in nonrhizosphere soil and rhizosphere soil and possessed a more complex microbial network structure compared with the cultivated variety Akihime. Genera such as *Bradyrhizobium* and *Anaeromyxobacter*, which are associated with nitrogen fixation and ammonification, and *Conexibacter*, which is associated with ecological toxicity resistance, exhibited higher relative abundances in the rhizosphere and nonrhizosphere soil samples of *F. nilgerrensis* compared with those of Akihime. Meanwhile, the ammonia-oxidizing archaea *Candidatus Nitrososphaera* and *Candidatus Nitrocosmicus* showed the opposite tendencies. We also found that the relative abundances of potential pathogenic genera and biocontrol bacteria in the Akihime samples were higher than those in the *F. nilgerrensis* samples. The relative abundances of *Blastococcus, Nocardioides, Solirubrobacter,* and *Gemmatimonas*, which are related to pesticide degradation, and genus Var*iovorax*, which is associated with root growth regulation, were also significantly higher in the Akihime samples than in the *F. nilgerrensis* samples. Moreover, the root endophytic microbiomes of both strawberry species, especially the wild *F. nilgerrensis*, were mainly composed of potential biocontrol and beneficial bacteria, making them important sources for the isolation of these bacteria. This study is the first to compare the differences in nonrhizosphere and rhizosphere soils and root endogenous microorganisms between wild and cultivated strawberries. The findings have great value for the research of microbiomes, disease control, and germplasm innovation of strawberry.

## 1 Introduction

Throughout their life cycle, plants constantly recruit microorganisms from the rhizosphere, phyllosphere, endosphere, and even from humans (Abdullaeva et al., 2021), and form a symbiotic functional body (Holobiont) with the fungi, prokaryotes, and viruses they have recruited (Hardoim et al., 2015; Hassani et al., 2018; Trivedi et al., 2020; Santoyo, 2022). The “holobiont” plays a crucial role in plant growth and reproduction; epiphytic and endophytic microorganisms contribute to promoting plant growth and resistance against pathogen invasion (De Tender et al., 2016; Zope et al., 2019). Beneficial microorganisms, whether of plant or nonplant origin, have been widely applied in agricultural production. At present, biological control has been widely used in the prevention and control of strawberry diseases. Relative studies have found that beneficial microorganisms such as some fungal groups [including *Trichoderma harzianum* and *Arbuscular mycorrhizal fungi* (AMF)], bacterial groups (*Bacillus subtilis* and *Pseudomonas fluoro*), and microbial organic fertilizers can improve the soil microbial environment, promote plant growth, enhance disease resistance and increase fruit yield (Hautsalo et al., 2016; Liu et al., 2021; Youseif et al., 2021; Li et al., 2022a). In particular, two bacterial populations, *Bacillus* and *Pseudomonas*, have attracted much attention because of their abilities to inhibit pathogen development and promote plant growth (Xu et al., 2015). Understanding the compositions and structures of microbial communities in the soils and roots of strawberries and the microbiome differences between disease-resistant and susceptible plants is important in obtaining pure cultures of pathogens and subsequently selecting beneficial microorganisms (Chung et al., 2020; van Ruijven et al., 2020; Yan et al., 2023).

*F. nilgerrensis* is a diploid strawberry widely distributed in central and southwest China. *F. nilgerrensis* has strong resistance to disease, drought and waterlogging; its fruit has special aroma, making it an excellent species resource for germplasm innovation of strawberry (Zhang et al., 2020; Cao et al., 2022; Hu et al., 2022). Akihime is one of the main varieties in Japan and was crossbred by Mr. Zhang Hong, a farmer breeder in Shizuoka Prefecture, Japan, in 1992. In 1996, Akihime was introduced to China by the Strawberry Research Institute of Donggang City, Liaoning Province, and successfully planted in China. Akihime is also the main cultivated variety in Yunnan, China, with a wide planting area and high yield, but poor storage and transportation resistance and susceptibility to powdery mildew (Kanto, 2009; Liu et al., 2014). With the expansion of greenhouse cultivation and continuous cropping, strawberries have been remarkably affected by pathogenic microbial infections, resulting in substantial losses (Husaini and Neri, 2016). However, field investigation did not reveal the pathological symptoms of *F. nilgerrensis*. We believe that the key of *F. nilgerrensis* to maintaining health in the wild may provide useful insights for the disease control of cultivated strawberries. Therefore, elucidating the differences in the microbiomes of the wild species, *F. nilgerrensis*, and the cultivated species, Akihime, is of great importance. However, to date, the differences in root-associated microbes and endophytes between these two species remain unknown.

As reported, wild plants have been found to harbor a higher abundance of beneficial bacteria and a greater microbial diversity in the rhizosphere compared to cultivated species, providing the former with stronger adaptability and disease resistance (Ma et al., 2019; Shi et al., 2019). When plants undergo artificial hybridization and domestication, the composition, structure, and function of the rhizosphere microbiome may change (Shenton et al., 2016; Tian et al., 2020). Furthermore, the use of pesticides and herbicides can lead to alterations in the composition and function of microbial communities (Pantigoso et al., 2020). On this basis, we postulated that there are differences in the microbial communities between *F. nilgerrensis* and Akihime, and formulated this study to explore further. With the rapid development of high-throughput sequencing and bioinformatics, culture-free methods provide a convenient and fast way to study the composition, structure, and function of plant microorganisms (Müller et al., 2016). To date, the microbiome of *F. nilgerrensis* has never been reported. Hence, high-throughput sequencing was used to compare the microbial communities of the wild species, *F. nilgerrensis*, and the cultivated strawberry, Akihime. We aimed to investigate the compositions, structures, and differences of microbial communities between the wild species (*Fragaria nilgerrensis*) and the cultivated species (*Fragaria × ananassa* Duch. cv. Akihime). The findings provide theoretical guidance and data support for the biological control of strawberry diseases and the isolation of culturable microorganisms, especially growth-promoting and disease-resistant strains.

## 2 Materials and methods

### 2.1 Sample collection

**Figure 1:**
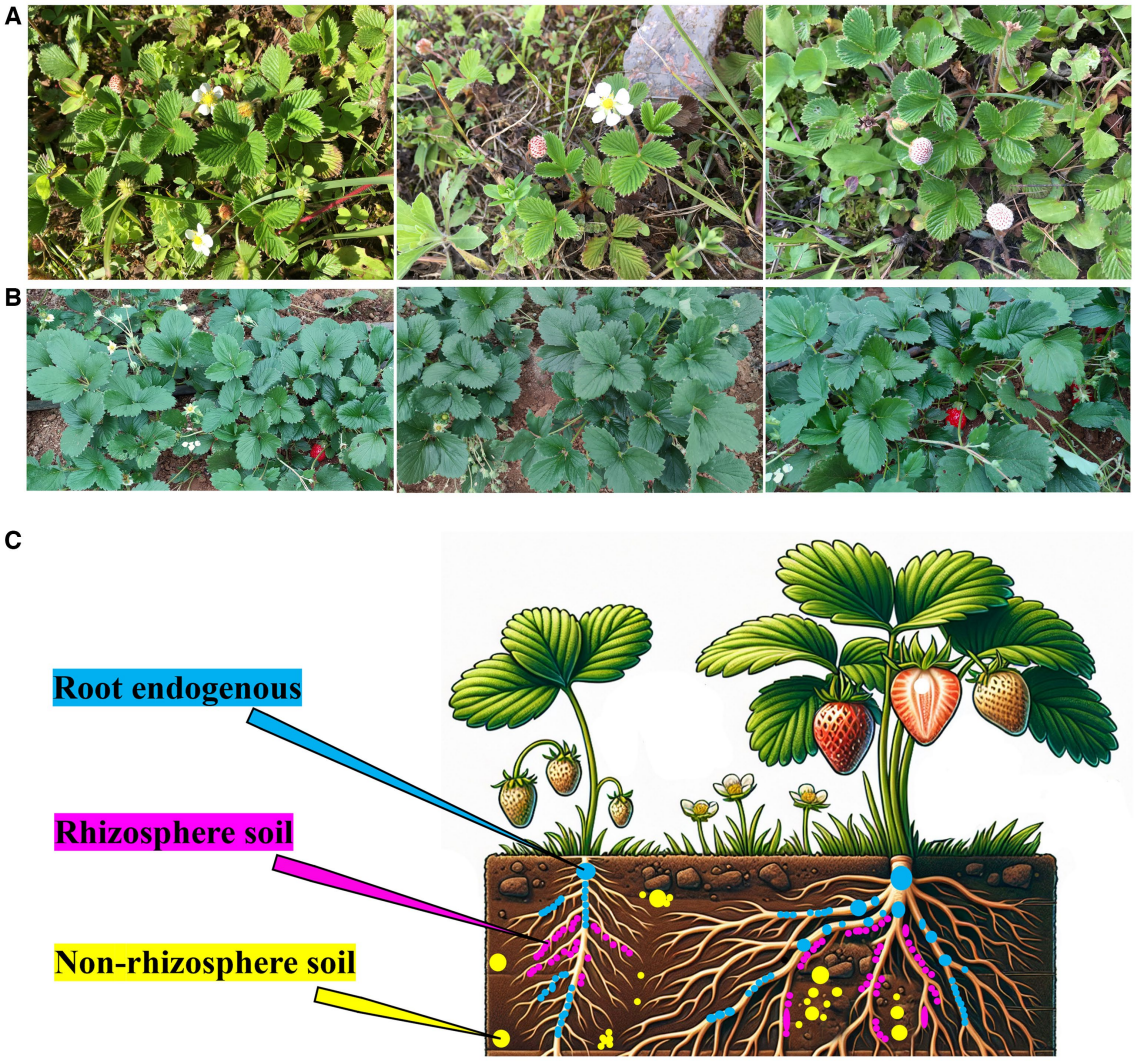
The natural habitat and morphology of *F. nilgerrensis* **(A)**, the growth conditions of Akihime in the greenhouse **(B)**, the sampling locations of Root endogenous, Rhizosphere soil, and Non-rhizosphere soil samples **(C)**.

Healthy *F. nilgerrensis* plants were collected from Kunming, Yunnan Province from May to July 2021 (102.24′30″ E; 25.06′57’ N; Altitude: 2138.7 m) (Figure 1A). Healthy Akihime plants were collected from Kunming, Yunnan Province (102.52′02″ E, 25.13′ 07 ‘48” N, altitude: 2107.0 m) (Figure 1B). The distance between the two places was approximately 79.94 km, the climate type and soil type were similar, and the collected Akihime and *F. nilgerrensis* specimens were in the flowering and fruiting stages. The sampling locations of the strawberry samples are shown in Figure 1C. Three different locations (as three replicate samples) were randomly selected for sampling using a 5-point sampling method, in which five strawberry plants were collected at each sampling location. The collection methods for samples from different parts were as follows. Nonrhizosphere soil: Plants with consistent age and height growth were randomly selected. First, the dead branches, fallen leaves, and topsoil around the plants were removed, and 0–20 cm^3^ of soil around the plants was then collected (Leff et al., 2015). After the collection was complete, the samples were mixed evenly and then packed in sterile bags. The *F. nilgerrensis* samples were labeled as F_nonrhizosphere soil, and the Akihime samples were labeled as A_nonrhizosphere soil. Rhizosphere soil: The large clumps of soil around the roots were removed, and the roots were gently shaken to dislodge loose soil. With a sterile brush, approximately 2 mL of soil was collected from the root surface and placed in a 50 mL sterile centrifuge tube while removing any remaining root fragments and fungal residues (Coleman-Derr et al., 2016). The *F. nilgerrensis* samples were labeled as F_rhizosphere soil, and the Akihime samples were labeled as A_rhizosphere soil. The method for collecting root tissue and extracting endophytic microorganisms from roots was as follows: strawberry roots were collected along with the soil samples, and the root tissues were surface-sterilized by sequentially washing them with sterile water for 30 s and 70% sterile ethanol for 2 min, soaking in 2.5% NaClO for 5 min, and immersing in sterile 70% ethanol for 30 s. Finally, the plant tissues were rinsed three times with sterile water to obtain surface-sterilized samples for extracting root endophytic microbial DNA (Bulgarelli et al., 2012; Edwards et al., 2015; Coleman-Derr et al., 2016). The *F. nilgerrensis* samples were labeled F_root endogenous, and the Akihime samples were labeled A_root endogenous (Figure 1C).

### 2.2 DNA extraction, PCR amplification, and sequencing

DNA was extracted from strawberry rhizosphere and nonrhizosphere soil samples using the PowerSoil DNA Isolation Kit (Mo Bio Laboratories, San Diego, CA, United States). Approximately 60–100 mg of the samples were weighed out into 2 mL sterile EP tubes, and DNA was extracted following the instructions of the kit. For strawberry root endosphere microbial DNA, extraction was performed using the HP Plant DNA Kit (OMEGA). Approximately 60–80 mg of the surface-sterilized root samples were weighed out into 1.5 mL sterile EP tubes, and DNA was extracted following the kit’s protocol (Yang et al., 2020; Su et al., 2021, 2022).

The purified DNA from each sample was used as the amplification template, and the V3–V4 region of the 16SrRNA gene of prokaryotes was amplified using the prokaryotic universal forward primer 341F (5′-CCTAYGGGRBGCASCAG-3′) and the reverse primer 806R (5′-GGACTACNNGGGTATCTAAT-3′) (Takahashi et al., 2014). The ITS2 gene of fungal groups was amplified using the universal forward primer ITS3F (5′-GCATCGATGAAGAACGCAGC-3′) and the reverse primer ITS4R (5′-TCCTCCGCTTATTGATATGC-3′) (Tedersoo et al., 2015). Following PCR amplification, the PCR products were detected using 1.5% agarose gel electrophoresis. The quality of sample DNA was assessed by employing the NanoDrop2000 instrument at the Public Experimental Platform of Yunnan Microbial Institute, ensuring that the OD_260_/OD_280_ ratio of each sample falls within the range of 1.8–2.0 with a concentration exceeding 30 ng/μL. For unqualified samples, DNA must be reextracted (Yang et al., 2020; Su et al., 2021, 2022). All the samples were submitted for sequencing on the Illumina MiSeq PE 300 platform at Majorbio Bio-Pharm Technology Co., Ltd. in Shanghai, China.

### 2.3 Bioinformatics analysis and data processing

After the sequencing was completed, all the raw data were subjected to paired-end sequence assembly and quality control using FLASH (V.1.2.11) (Magoc and Salzberg, 2011) and Fastp (V.0.19.6) (Chen et al., 2018) to generate clean tags. Subsequently, Uparse (V. 11) (Edgar, 2013), Usearch (Edgar, 2010), and Qiime (V.1.9.1) (Kuczynski et al., 2011) were then utilized for operational taxonomic unit (OTU) selection, counting, and sequence clustering at a 97% similarity threshold. The RDP classifier software (Hördt et al., 2020) was applied for species annotation based on the representative sequences of OTUs, with the threshold set at 0.7. Bacterial taxa were aligned against the Silva database (https://www.arb-silva.de/, V silva138/16 s), and fungal taxa were aligned against the UNITE database (https://unite.ut.ee/, V unite8.0/its_fungi).

During data analysis, all the samples were normalized based on the sequence count of the sample with the lowest sequencing volume. And, the mitochondrial and chloroplast sequences associated with the plant host were excluded. Subsequently, bioinformatics analyses and graphic visualization, which included Alpha diversity analysis [utilizing Mothur (v1.30.2) (Schloss et al., 2009)], microbiome community analysis (encompassing species composition analysis, species differential analysis), PLS-DA analysis, and single-factor correlation network analysis [performed with Gephi (Jacomy et al., 2014) and network (https://networkx.org/)], were then conducted on the I-Sanger platform (Majorbio Bio-Pharm Technology Co., Ltd., Shanghai, China; www.i-sanger.com). SPSS V.26 (SPSS Inc., Chicago, Ill., United States) and Excel 2021 (Microsoft) were also used for analysis.

## 3 Results

### 3.1 Statistics of sequencing results and analysis of microbial diversity and richness

In this study, 18 samples from nonrhizosphere soil, rhizosphere soil, and root of *F. nilgerrensis* and Akihime were sequenced. After low-quality data and sequences related to mitochondria and chloroplasts were filtered out, valid bacterial and fungal sequences were obtained. A total of 364,992 sequences were obtained from the Akihime samples, which is lower than the 442,176 sequences obtained from the *F. nilgerrensis* samples. After sequence clustering and annotation, the OTUs for bacteria and fungi were obtained (Supplementary Table S1). Analysis of OTU data revealed that the fungal and bacterial OTU numbers in *F. nilgerrensis* samples were higher than those in Akihime, with the number of fungal OTUs significantly higher in the *F. nilgerrensis* samples than in the Akihime samples (*p* < 0.05) (Supplementary Table S1). Sequencing results showed that the effective data coverage after screening exceeded 95%, indicating that the data were sufficient for subsequent analyses (Supplementary Table S1).

At a 97% similarity threshold, the Shannon index was calculated for each sample to generate dilution curves. The results showed that the dilution curve tended to be gentle, indicating that the sequencing data reached saturation and effectively covered the majority of microbial communities in each sample (Supplementary Figure S1) and consequently made the results reliable. To clarify the abundances and diversities of the bacterial and fungal communities in the strawberry root microenvironment, this study calculated alpha and beta diversity indices, including Shannon index and Simpson index for characterizing community diversity and Ace index, Chao index, and Sobs index for characterizing community richness. The statistical results of fungal community diversity indices showed that the diversities and richnesses of the fungal communities in the rhizosphere soil, nonrhizosphere soil, and roots of *F. nilgerrensis* were higher than those of Akihime and the differences were significant (*p* < 0.05) According to the statistical results of bacterial community diversity indices, the richness and diversity of bacterial communities in the rhizosphere soil and nonrhizosphere soil were higher for *F. nilgerrensis* than for Akihime, but the differences were not significant. The root endosphere bacteria had higher richness but lower diversity in *F. nilgerrensis* than in Akihime, and the differences were significant (*p* < 0.05) (Table 1). In addition, this study compared the richness and diversity of microbial communities in the nonrhizosphere soil, rhizosphere soil, and roots of *F. nilgerrensis* and Akihime. The richness and diversity of the bacterial and fungal communities in the nonrhizosphere soil and rhizosphere soil samples of *F. nilgerrensis* and Akihime were significantly higher than those in their root endosphere samples (*p* < 0.05). The diversities of bacterial and fungal communities in the root microenvironments of *F. nilgerrensis* and Akihime were in the following order: rhizosphere soil > nonrhizosphere soil > intraroot (Supplementary Table S2).

**Table 1:** Comparison of *α* diversity indices of bacterial and fungal communities in different samples.

Amplification region	Sample names	Community diversity		Community richness		
		Shannon	Simpson	Ace	Chao	Sobs
16S	A_Non_rhizosphere soil	6.623 ± 0.03567a	0.00353 ± 0.000138a	3472.601 ± 53.308a	3436.881 ± 85.732a	2539.667 ± 22.811a
	F_Non_rhizosphere soil	6.723 ± 0.03974b	0.00345 ± 0.000340a	3569.328 ± 140.850a	3566.978 ± 237.504a	2660.667 ± 56.083b
	A_rhizosphere soil	6.671 ± 0.0514a	0.00290 ± 0.000273a	3234.718 ± 6.834a	3197.135 ± 14.847a	2327.333 ± 27.538a
	F_rhizosphere soil	6.760 ± 0.2197a	0.00391 ± 0.001534a	3572.441 ± 277.268a	3550.256 ± 253.285a	2567.667 ± 223.241a
	A_ Root endogenous	2.987 ± 0.0846a	0.09055 ± 0.007033a	248.538 ± 92.245a	194.893 ± 56.144a	111.333 ± 5.686a
	F_ Root endogenous	2.414 ± 0.0256b	0.19463 ± 0.00222b	488.949 ± 166.227a	320.794 ± 91.527a	124.000 ± 3.464b
ITS2	A_Non_rhizosphere soil	3.630 ± 0.0287a	0.09505 ± 0.003347a	826.550 ± 21.1332a	827.842 ± 22.899a	662.333 ± 9.452a
	F_Non_rhizosphere soil	4.949 ± 0.0131b	0.03523 ± 0.000729b	1925.258 ± 28.521b	1915.721 ± 53.372b	1534.333 ± 21.362b
	A_rhizosphere soil	3.909 ± 0.1699a	0.04791 ± 0.006984a	834.840 ± 51.576a	829.853 ± 47.579a	736.667 ± 51.394a
	F_rhizosphere soil	4.967 ± 0.0868b	0.03121 ± 0.003435b	2187.663 ± 56.286b	2194.218 ± 39.893b	1752.333 ± 64.049b
	A_ Root endogenous	0.884 ± 0.0232a	0.55765 ± 0.006161a	107.000 ± 29.135a	91.833 ± 25.342a	63.000 ± 4.359a
	F_ Root endogenous	2.326 ± 0.1143b	0.38671 ± 0.028888b	622.714 ± 18.071b	617.057 ± 27.908b	510.667 ± 12.702b

A_, Akihime sample; F_, Fragaria nilgerrensis (M ± SD, *n* = 3). *T* values are based on independent sample *t*-tests. Significance is indicated by different letters.

Partial Least Squares Discriminant Analysis (PLS-DA) showed that the *F. nilgerrensis* and Akihime samples could be distinguished and clustered into two groups, indicating differences in their microbial compositions. According to the scatter distribution of results, the compositions of microorganisms significantly differed among the samples of *F. nilgerrensis* or Akihime (Supplementary Figure S2).

### 3.2 Compositions and structures of bacterial and fungal communities

#### 3.2.1 Composition and structure of bacterial community

**Figure 2:**
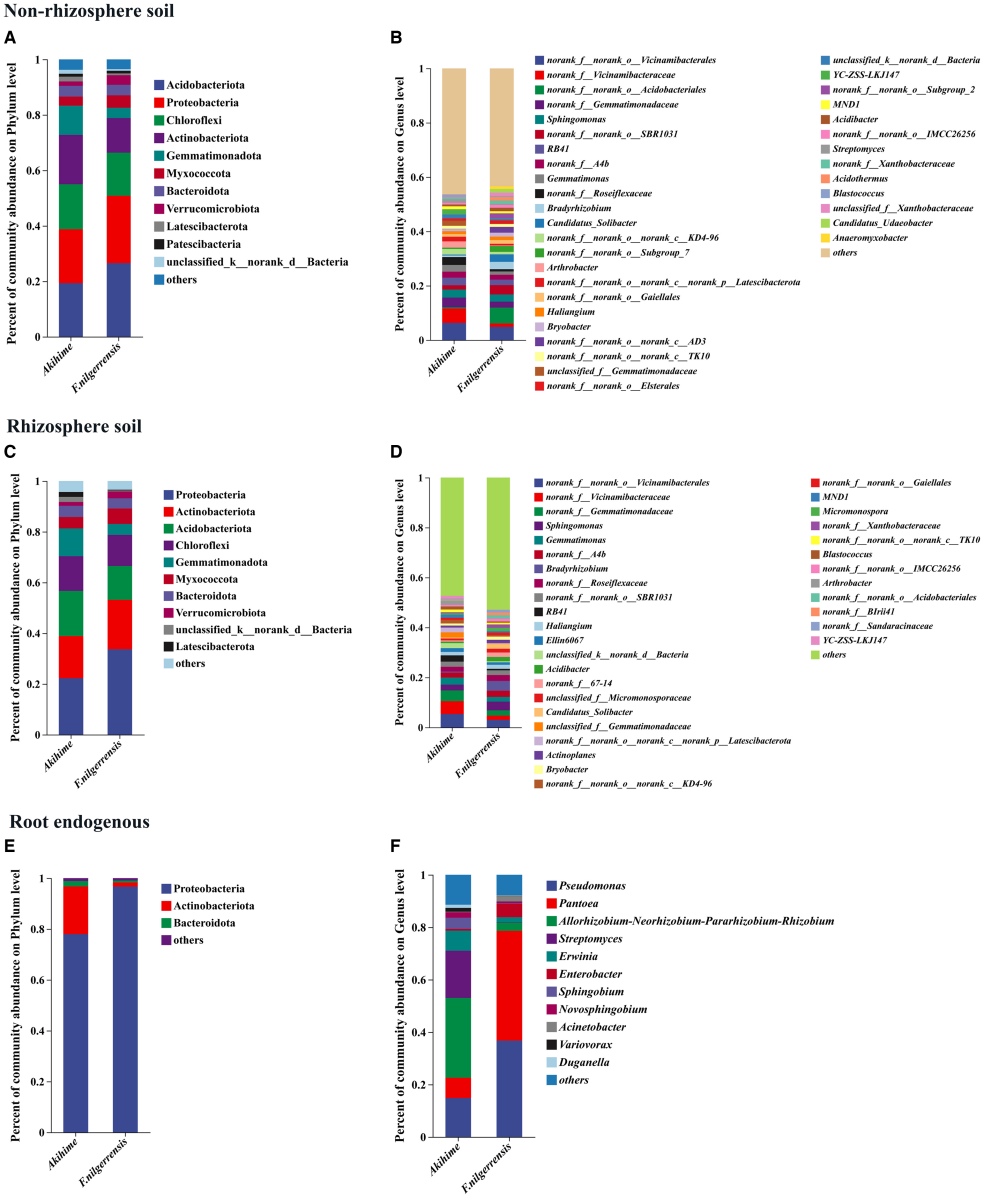
Relative abundances of bacterial community compositions at the phylum **(A,C,E)** and genus **(B,D,F)** levels are determined in different samples (*n* = 3). The genus and phylum with relative abundance <1% is combined into“others”.

For bacterial community, a total of 36 phyla, 113 classes, 288 orders, 449 families, 895 genera, and 5,915 OTUs were identified. At the phylum level, 11, 10, and 3 bacterial phyla with relative abundances of more than 1% were detected in the nonrhizosphere soil, rhizosphere soil, and root endosphere samples, respectively. *Actinobacteriota*, *Bacteroidota*, and *Proteobacteria* were the common taxa in the *F. nilgerrensis* and Akihime samples. *Acidobacteriota*, *Actinobacteriota*, *Proteobacteria*, *Chloroflexi*, and *Gemmatimonadota* were the most abundant phyla in the rhizosphere and nonrhizosphere soil samples of both species, and *Proteobacteria* was the most abundant phylum in the root endosphere samples (Figures 2A,C,E). Moreover, the relative abundances of *Patescibacteria* in the nonrhizosphere soil samples of *F. nilgerrensis* and Akihime were more than 1% (Figure 2A).

At the genus level, 36, 34, and 11 genera with relative abundances of more than 1% were detected in the nonrhizosphere soil, rhizosphere soil, and root endosphere samples, respectively. Among them, *Sphingomonas, RB41, Gemmatimonas, Bradyrhizobium, Candidatus_Solibacter, Arthrobacter, Haliangium, Bryobacter, YC-ZSS-LKJ147, Acidibacter, Blastococcus, MND1*, and *no rank* and *unclassified* taxa were abundant in the nonrhizosphere soil and rhizosphere soil samples of *F. nilgerrensis* and Akihime (Figures 2B,D). The relative abundances of *YC-ZSS-LKJ147* in the nonrhizosphere soil and rhizosphere soil samples of Akihime were more than 1%, which were 1.92 and 1.06%, respectively (Figures 2B,D). The relative abundance of *Candidatus_Udaeobacter* in the nonrhizosphere soil samples of *F. nilgerrensis* was more than 1%, which was 1.3% (Figure 2B). The abundances of *Bradyrhizobium* in the nonrhizosphere and rhizosphere soil samples of *F. nilgerrensis* (2.7 and 3.94%) were higher than those in the Akihime samples (0.57 and 0.46%), and the relative abundance of *Blastococcus* in the Akihime samples (1.07, 1.24%) was higher than that in the *F. nilgerrensis* samples (0.38 and 0.52%) (Figures 2B,D). In the root endosphere samples, the relative abundances of *Pseudomonas, Pantoea, Enterobacter*, and *Acinetobacter* were higher in *F. nilgerrensis* (36.79, 41.87, 5.13, and 2.09%) than in Akihime (14.83, 7.76, 0.65, and 0.2%). Meanwhile, the relative abundances of *Allorhizobium-Neorhizobium-Pararhizobium-Rhizobium, Streptomyces, Erwinia, Sphingobium, Novosphingobium,* and Var*iovorax* in the Akihime samples (30.44, 18.01, 7.69, 4.29, 2.07, and 1.46%) were higher than those in the *F. nilgerrensis* samples (3.11, 0.15, 1.9, 0.26, 0.61, and 0.12%) (Figure 2F).

#### 3.2.2 Composition and structure of fungal community

**Figure 3:**
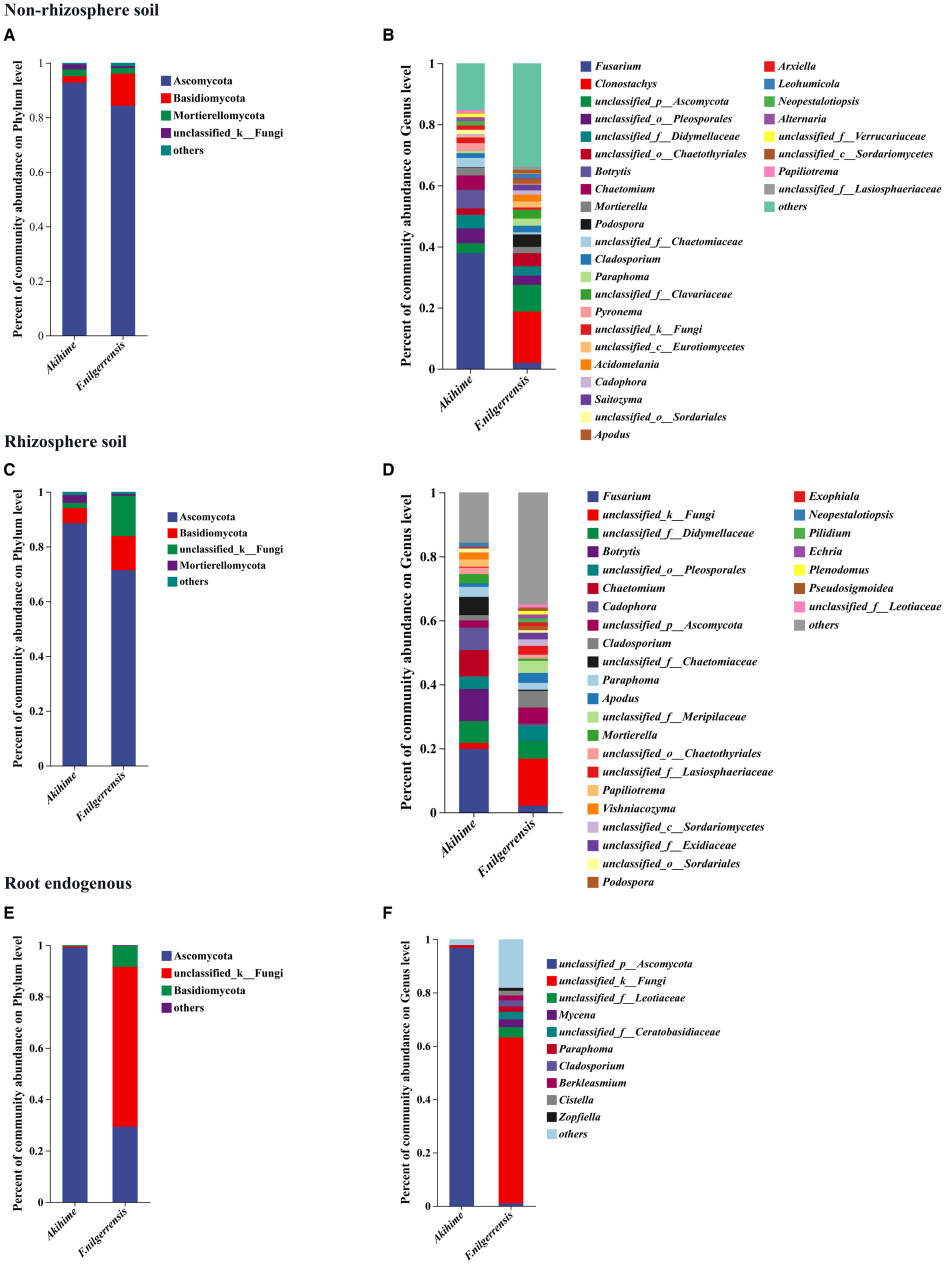
Relative abundances of fungal community compositions at the phylum **(A,C,E)** and genus **(B,D,F)** levels are determined in different samples (*n* = 3). The genus and phylum with relative abundance <1% is combined into“others”.

A total of 16 phyla, 63 classes, 163 orders, 365 families, 855 genera, and 3,620 OTUs of fungal communities were identified from the samples of *F. nilgerrensis* and Akihime. At the phylum level, 4, 4, and 3 phyla with relative abundance exceeding 1% were detected in the nonrhizosphere soil, rhizosphere soil, and root endosphere samples, respectively. Among them, *Ascomycota*, *Basidiomycota*, and *unclassified_k__Fungi* were the common taxa in both strawberry species, and *Ascomycota* was the main group in the samples of *F. nilgerrensis* and Akihime (Figures 3A,C,E). The main group in the root endosphere samples of Akihime was *Ascomycota* with a relative abundance of 99.08%, and the main groups in the root endosphere samples of *F. nilgerrensis* were *Ascomycota* and *unclassified_k__Fungi* with relative abundances of 29.3 and 62.2%, respectively (Figure 3E).

At the genus level, 30, 29, and 10 genera were detected in the nonrhizosphere soil, rhizosphere soil, and root endosphere samples of both species, respectively. *Fusarium, Mortierella, Cladosporium, Paraphoma*, and *Cadophora* were the most abundant taxa in the nonrhizosphere soil and rhizosphere soil samples of *F. nilgerrensis* and Akihime (Figures 3B,D). The taxa with high relative abundances in the nonrhizosphere soil samples of *F. nilgerrensis* were *Fusarium, Clonostachys, Mortierella, Cladosporium, Paraphoma, Fusarium, Acidomelania, Saitozyma, podospora* and *Apodus*, and those in the nonrhizosphere soil samples of Akihime were *Fusarium, Botrytis, Chaetomium, Mortierella, Cladosporium, Pyronema, Neopestalotiopsis*, and *Alternaria* (Figure 2B). The taxa with high relative abundances in the rhizosphere soil samples of *F. nilgerrensis* were *Cladosporium, Paraphoma, podospora, Apodus, Exophiala, Fusarium, Pilidium, Echria, Plenodomus*, and *Pseudosigmoidea*, and those in the rhizosphere soil samples of Akihime were *Fusarium, Botrytis, Chaetomium, Cadophora, Cladosporium, Pyronema, Apodus, Neopestalotiopsis, Papiliotrema*, and *Vishniacozyma* (Figure 3D). In the root endosphere samples, the relative abundance of *unclassified_p__Ascomycota* was more than 96% in the Akihime samples, and that of *unclassified_k__Fungi* was more than 62% in the *F. nilgerrensis* samples (Figure 2F). *Mycena, Berkleasmium, Cistella*, and *Zopfiella* were also the groups with high relative abundance in the root endosphere samples of *F. nilgerrensis* (Figure 3F).

### 3.3 Analysis of differences In microbial composition and structure

Based on the abundance data of bacterial and fungal communities, Welch’s t-test was used to detect the taxa with different abundances among the microbial communities. The significance of the difference was evaluated by hypothesis tests.

#### 3.3.1 Analysis of bacterial community differences

**Figure 4:**
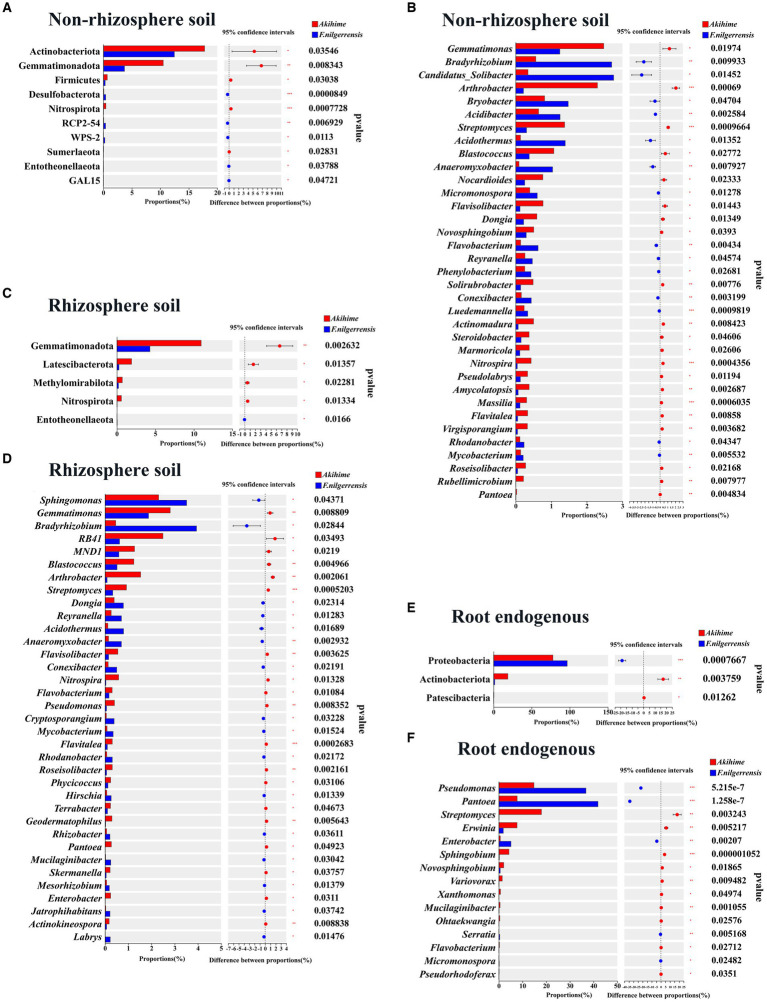
Analysis of differences in the relative abundance of bacterial communities in different samples (*n* = 3) at phylum **(A,C,E)** and genus **(B,D,F)** levels.

At the phylum level, 11 phyla groups of the nonrhizosphere soil samples showed significant differences, among them, the relative abundances of *Acidobacteriota*, *Gemmatimonadota*, *Firmicutes, Nitrospirota, Sumerlaeota,* in the Akihime samples were significantly higher than those in the *F. nilgerrensis* samples (*p* < 0.05), while the relative abundances of *Desulfobacterota*, *RCP2-54*, *WPS-2*, *Entotheonellaeota* and *GAL15* were significantly lower than those of *F. nilgerrensis* samples (*p* < 0.05) (Figure 4A). 6 phyla groups of the rhizosphere soil samples showed significant differences, among them, the relative abundances of *Gemmatimonadota*, *Latescibacterota*, *Methylomirabilota*, and *Nitrospirota* in the Akihime samples were significantly higher than those in the *F. nilgerrensis* samples (*p* < 0.05), while the relative abundance of *Entotheonellaeota* was significantly lower than that in the *F. nilgerrensis* samples (*p* < 0.05) (Figure 4C). 3 phyla groups that in the root endosphere samples showed significant differences, among them, the relative abundances of *Actinobacteriota* and *Patescibacteria* in the Akihime samples was significantly higher than that in the *F. nilgerrensis* samples (*p* < 0.05), while the relative abundance of *Proteobacteria* was significantly lower than that in the *F. nilgerrensis* samples (*p* < 0.05) (Figure 4E).

At the genus level, 244 genera of the nonrhizosphere soil samples showed significant differences. In particular, the relative abundances of *Bradyrhizobium, Candidatus_Solibacter, Bryobacter, Acidibacter, Acidothermus, Anaeromyxobacter, micromonospora, Flavisolibacter, Reyranella*, and *conexibacter* in the *F. nilgerrensis* samples were significantly higher than those in the Akihime samples (*p* < 0.05). Meanwhile, *Gemmatimonas, Arthrobacter, Streptomyces, Blastococcus, nocardioides, Flavisolibacter,* and *Pantoea* showed significantly lower relative abundances in the *F. nilgerrensis* samples (*p* < 0.05) (Figure 4B). A total of 168 genera of the rhizosphere soil samples showed significant differences. *Sphingomonas, Bradyrhizobium, Dongia, Reyranella, Acidothermus, Anaeromyxobacter,* and *conexibacter* showed significantly higher relative abundances in the *F. nilgerrensis* samples than in the Akihime samples (*p* < 0.05). Meanwhile, genera such as *Gemmatimonas, Blastococcus, Arthrobacter, Streptomyces, Flavisolibacter, Pseudomonas*, and *Pantoea* showed significantly lower abundances in the *F. nilgerrensis* samples than in the Akihime samples (*p* < 0.05) (Figure 4D). For the root endosphere samples, differences were observed in the relative abundance of 18 genera. Among them, the relative abundances of *Pseudomonas, Pantoea,* and *Enterobacter* were significantly higher in the *F. nilgerrensis* samples than in the Akihime samples (*p* < 0.05), and those of *Streptomyces, Erwinia, Sphingobium,* Var*iovorax*, and *Xanthomonas* were significantly higher in the Akihime samples than in the *F. nilgerrensis* samples (*p* < 0.05) (Figure 4F).

#### 3.3.2 Analysis of fungal community differences

**Figure 5:**
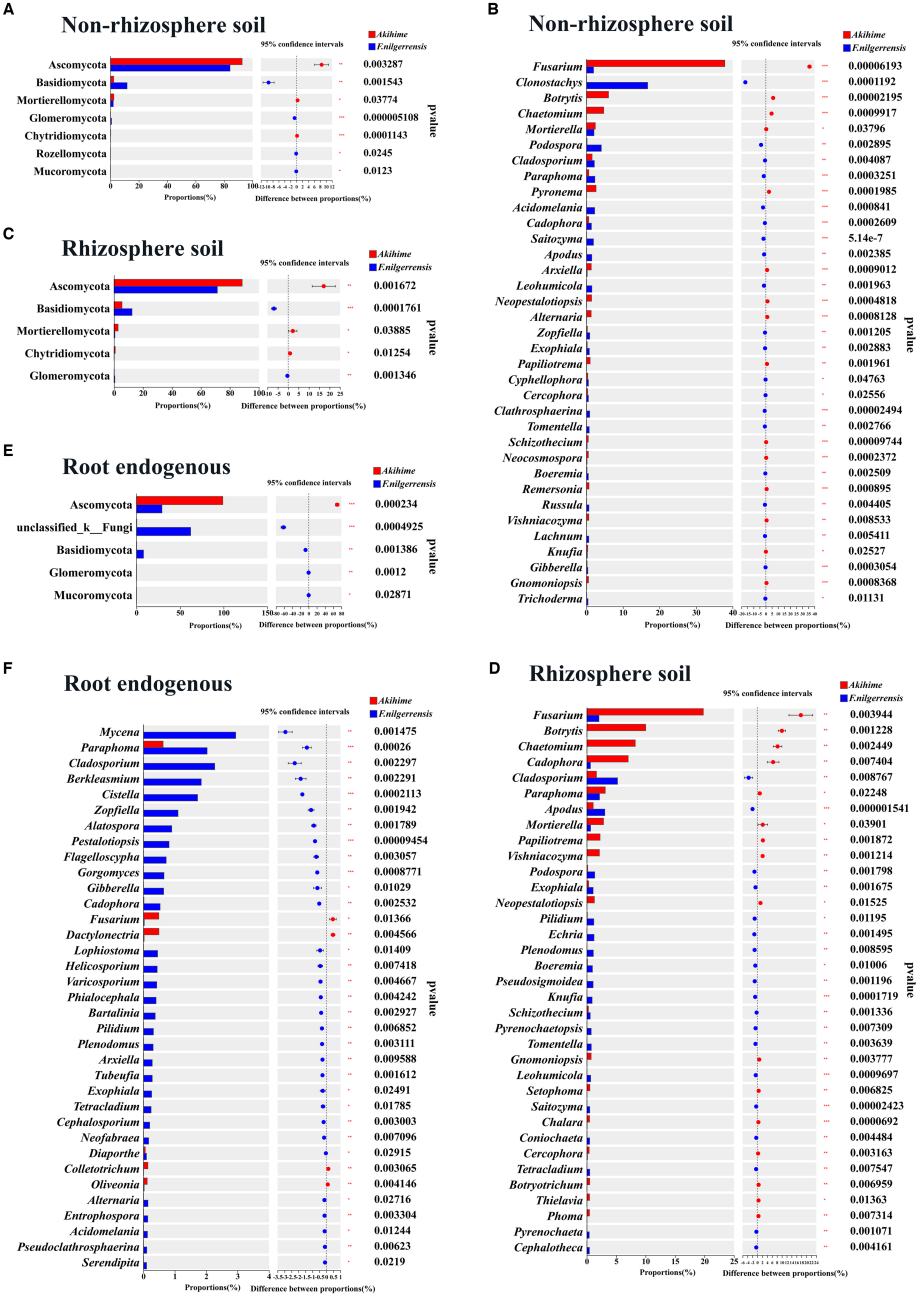
Analysis of differences in the relative abundance of fungal communities in different samples (*n* = 3) at phylum **(A,C,E)** and genus **(B,D,F)** levels.

At the phylum level, nine phyla in the nonrhizosphere soil showed different relative abundances between the two species. *Basidiomycota, Glomeromycota, Rozellomycota,* and *Mucoromycota* showed significantly higher relative abundance in the *F. nilgerrensis* samples than in the Akihime samples (*p* < 0.05), and *Ascomycota* and *Mortierellomycota* had significantly higher relative abundance in the Akihime samples than in the *F. nilgerrensis* samples (*p* < 0.05) (Figure 5A). Seven phyla of the rhizosphere soil samples showed significant differences in their relative abundances. Among them, the relative abundances of *Basidiomycota* and *Glomeromycota* in the *F. nilgerrensis* samples were significantly higher than those in the Akihime samples (*p* < 0.05), and the relative abundances of *Ascomycota*, *Mortierellomycota*, and *Chytridiomycota* in the Akihime samples were significantly higher than those in the *F. nilgerrensis* samples (*p* < 0.05) (Figure 5C). Five phyla of the root endosphere samples showed significant differences in their relative abundances. Among them, the relative abundances of *Basidiomycota*, *Glomeromycota*, *mucoromycota*, and *Mucoromycota* in the *F. nilgerrensis* samples were significantly higher than those in the Akihime samples, and that of *Ascomycota* in the Akihime samples was significantly higher than that in the *F. nilgerrensis* samples (*p* < 0.05) (Figure 5E).

At the genus level, 312 genera in the nonrhizosphere soil showed differences in their relative abundances. Among them, *Clonostachys, Podospora, Cladosporium, Paraphoma, Acidomelania, Saitozyma, Cadophora, Apodus,* and *Leohumicola* showed significantly higher relative abundances in the *F. nilgerrensis* samples than in the Akihime samples (*p* < 0.05), and the relative abundances of *Fusarium, Botrytis,* and *Pyronema* in the Akihime samples were significantly higher than those in the *F. nilgerrensis* samples (*p* < 0.05) (Figure 5B). A total of 313 genera of the rhizosphere soil samples showed significant differences. Among them, the relative abundances of *Cladosporium, Apodus, podospora, Exophiala*, *Echria, Pilidium, Plenodomus, Pseudosigmoidea, Boeremia, Knufia, Pyrenochaetopsis,* and *Tomentella* were significantly higher in the *F. nilgerrensis* samples than in the Akihime samples (*p* < 0.05), and those of *Fusarium, Botrytis, Chaetomium, Cladosporium, Cadophora, Papiliotrema, mortierella*, *Vishniacozyma*, and *Neopestalotiopsis* in the Akihime samples were significantly higher than those in the *F. nilgerrensis* samples (*p* < 0.05) (Figure 5D). A total of 138 genera of the root endosphere samples showed significant differences. Among them, the relative abundances of *Mycena, Paraphoma, Cladosporium, Berkleasmium, Cistella, Zopfiella, Alatospora, Pestalotiopsis, Flagelloscypha, Gorgomyces,* and *Helicosporium* were significantly higher in the *F. nilgerrensis* samples than in the Akihime samples (*p* < 0.05), and those of *Fusarium, Dactylonectria, Colletotrichum,* and *Oliveonia* in the Akihime samples were significantly higher than those in the *F. nilgerrensis* samples (*p* < 0.05) (Figure 5F).

### 3.4 Relative abundances and differences of pathogenic and beneficial taxa In different samples

**Figure 6:**
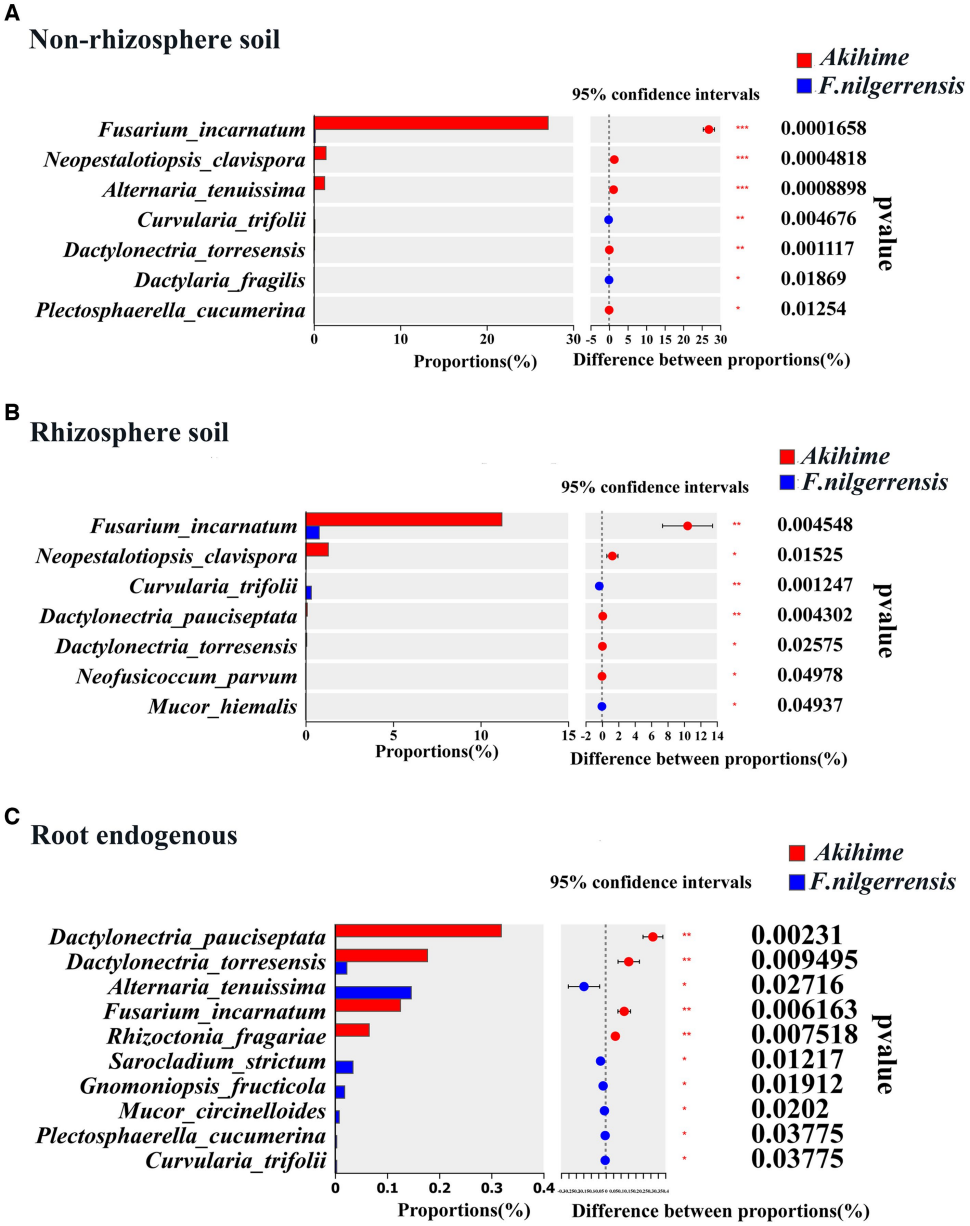
Analysis of relative abundance differences of fungal pathogen taxa in different samples (*n* = 3).

*Erwinia amylovora* can causes bacterial blight in strawberries (Öztürk and Soylu, 2022), and *Xanthomonas fragariae* can causes the angular leaf spot and crown rot of strawberries (Timilsina et al., 2020; Song et al., 2021). At the genus level, the relative abundances of *Erwinia* and *Xanthomonas* (Expert et al., 2004; Timilsina et al., 2020) in the root endosphere samples of Akihime were significantly higher than those in the *F. nilgerrensis* samples (*p* < 0.05) (Figure 4F). In addition, the *Rhodococcus fascians* taxa (APS), which can cause strawberry cauliflower disease, was detected in the rhizosphere soil and root endosphere samples of Akihime (data not shown). At the genus level, the relative abundances of potential fungal pathogens in the genera including *Fusarium* (Gwinn et al., 2022)*, Botrytis* (Drobek et al., 2021)*, Rhizoctonia* (Frąc et al., 2018; Pandit et al., 2022)*, Dactylonectria* (Su et al., 2022)*, Neopestalotiopsis* (Darapanit et al., 2021)*, Colletotrichum* (Sangiorgio et al., 2022), and *Cadophora* (Travadon et al., 2015) were different between the *F. nilgerrensis* and Akihime samples (Figure 5). For the rhizosphere soil samples, the relative abundances of *Fusarium, Botrytis, Neopestalotiopsis, Cadophora* in *F. nilgerrensis* samples was significantly higher than those in Akihime samples (*p* < 0.05) (Figure 5D). For the root endosphere samples, the relative abundances of *Dactylonectria*, *Colletotrichum, Fusarium* and *Rhizoctonia* in Akihime samples were significantly higher than those in *F. nilgerrensis* samples (*p* < 0.05) (Figure 5F). In addition, at least 14 fungal pathogen taxa were identified in the *F. nilgerrensis* and Akihime samples. For the nonrhizosphere soils samples, the relative abundances of Fungal pathogen taxa including *Fusarium incarnatum* (Ayoubi and Soleimani, 2016), *Neopestalotiopsis clavispora* (Gilardi et al., 2019) and *Alternaria tenuissima* (Fu et al., 2019) in Akihime samples were significantly higher than those in *F. nilgerrensis* samples (*p* < 0.05) (Figure 6A). For the rhizosphere soil samples, the relative abundances of *Fusarium incarnatum* and *Neopestalotiopsis clavispora* in Akihime samples were significantly higher than those in *F. nilgerrensis* samples (*p* < 0.05), while the relative abundance of *Curvularia trifolii* (Zhang et al., 2022b) was significantly lower than that in *F. nilgerrensis* samples (*p* < 0.05) (Figure 6B); For the root endosphere samples, the relative abundances of *Fusarium incarnatum, Rhizoctonia fragariae* (Erper et al., 2021), *Dactylonectria pauciseptata* and *Dactylonectria torresensis* (Chen et al., 2021) in the Akihime samples were significantly higher than in the samples of *F. nilgerrensis*, while the relative abundances of *Alternaria tenuissima, Gnomoniopsis fragariae* (Fang et al., 2011) were significantly lower than those in the *F. nilgerrensis* samples (Figure 6C).

*Micromonospora, Sphingomonas, Streptomyces, Pseudomonas*, and *Flavobacterium* (Hirsch and Valdes, 2010; Carrion et al., 2019; Asaf et al., 2020; Tariq et al., 2020; Alam et al., 2022; Collinge et al., 2022; Purtschert-Montenegro et al., 2022) are essential source of biocontrol bacteria. In this study, these genera were annotated and exhibited differences in relative abundance between the *F. nilgerrensis* and Akihime samples. For the nonrhizosphere soil samples, the relative abundance of *Streptomyces* in the Akihime samples was significantly higher than that in the *F. nilgerrensis* samples (*p* < 0.05), and those of *Micromonospora* and *Flavobacterium* in the Akihime samples were significantly lower than those in the *F. nilgerrensis* samples (*p* < 0.05) (Figure 4B). For the rhizosphere soil samples, except *Sphingomonas*, the relative abundances of *Streptomyces, Pseudomonas*, and *Flavobacterium* in the Akihime samples were significantly higher than those in the *F. nilgerrensis* samples (*p* < 0.05) (Figure 4D). For the root endosphere samples, the relative abundances of *Streptomyces* and *Flavobacterium* in the Akihime samples were significantly higher than those in the *F. nilgerrensis* samples (*p* < 0.05), and those of *Micromonospora* and *Pseudomonas* were significantly lower than those in the *F. nilgerrensis* samples (*p* < 0.05) (Figure 4F). *Acidibacter, Arthrobacter, Bradyrhizobium, Gemmatimonas, Nocardioides,* Var*iovorax, Blastococcus, Flavisolibacter, Anaeromyxobacter,* and *Enterobacter* (Kuypers et al., 2018; Zhang et al., 2019; Finkel et al., 2020; Kalam et al., 2020; Du et al., 2022; Zhang et al., 2022a; Li et al., 2022b) are important sources of beneficial bacteria and were annotated in both strawberry species with significant differences in relative abundance (*p* < 0.05) (Figure 4). *Chaetomium* is a potential source of biocontrol fungi (Moya et al., 2020). In this study, the relative abundance of *Chaetomium* in the nonrhizosphere and rhizosphere soil samples of Akihime was significantly higher than that in the *F. nilgerrensis* samples (*p* < 0.05) (Figures 5B,D). *Mortierella* is an important source of beneficial fungi (Büttner et al., 2021; Ozimek and Hanaka, 2021), and its relative abundance in the Akihime samples was significantly higher than that in the *F. nilgerrensis* samples (*p* < 0.05) (Figures 5B,D).

### 3.5 Correlation analysis of bacterial and fungal communities in different samples

The Spearman rank correlation coefficient was calculated for the genera in the top 50 total abundance in each sample to reflect the correlation (Barberán et al., 2014). We also conducted a single-factor correlation network analysis and calculated related parameters such as average degree, density, and modularity index (Table 2). The results showed that the interaction networks in the nonrhizosphere soil, rhizosphere soil, and root endosphere samples of *F. nilgerrensis* were more complex and connected than those in the Akihime samples (Table 2; Supplementary Figures 3, 4), suggesting that the microbiome of *F. nilgerrensis* is more stable than that of Akihime and is more conducive to adapt to the complex wild environment. Rhizosphere soil is a core region of plant-microbial interactions, a large number of microorganisms, such as bacteria, actinomycetes, fungi, and soil animals clustered around plant roots, showing specific physical, chemical, and biological properties (Zhou et al., 2022). In this study, we found that the complexity of microbial interactions in different samples showed the following complexity pattern: rhizosphere soil samples > nonrhizosphere soil samples > root endosphere samples (Table 2; Supplementary Figures 3, 4), which indicated that there were complex microbial interactions in the rhizosphere soil and verified the complexity of the rhizosphere soil.

**Table 2:** Univariate correlation network index of different samples.

Amplification region	Sample names	Nodes	Edges	Average degree	Density	Modularity index	Positive	Negative
16S	F_Non-rhizosphere soil	48	508	20.74	0.432	0.256	253	255
	A_Non-rhizosphere soil	50	426	16.71	0.334	0.585	294	132
	F_Rhizosphere soil	49	632	25.80	0.537	0.338	425	207
	A_Rhizosphere soil	50	432	16.94	0.339	0.527	225	207
	F_Root endogenous	50	204	8.16	0.167	0.778	99	105
	A_Root endogenous	50	230	9.20	0.188	0.700	124	206
ITS2	F_Non-rhizosphere soil	50	456	17.88	0.358	0.492	222	234
	A_Non-rhizosphere soil	49	382	15.28	0.312	0.644	189	193
	F_Rhizosphere soil	50	481	18.86	0.377	0.514	234	247
	A_Rhizosphere soil	48	387	15.48	0.316	0.494	215	172
	F_Root endogenous	49	406	16.24	0.331	0.591	313	93
	A_Root endogenous	49	191	7.64	0.156	0.791	125	66

A_, Akihime sample; F_, Fragaria nilgerrensis. The analysis is based on the genera with relative abundance of top 50.

### 3.6 Archaeal communities of wild and cultivated strawberries

In this report, we separately analyzed the archaeal communities in wild species (*F. nilgerrensis*) and cultivated strawberry (Akihime) samples. The results showed that there were differences in the compositions and structures of archaeal communities between *F. nilgerrensis* and Akihime. The archaea we detected belonged to 5 phyla, which were *Crenarchaeota*, *Halobacterota*, *Thermoplasmatota*, *Nanoarchaeota* and unclassified_d__Unclassified (Supplementary Figure S5). In the nonrhizosphere soil and rhizosphere soil samples of the two strawberry varieties, *Crenarchaeota* and *Thermoplasmatota* were the most abundant phyla (Supplementary Figures S5A,C) and *Candidatus_Nitrocosmicus* and *Candidatus_Nitrososphaera* were the most abundant genera (Supplementary Figure 5B,D). *Crenarchaeota* and unclassified_d__Unclassified were the most abundant group in the root endosphere samples of Akihime and *F. nilgerrensis*, respectively (Supplementary Figure 5E). Differential analysis showed that the relative abundance of *Crenarchaeota* significantly differed in the nonrhizosphere and rhizosphere soil samples of the two kinds of strawberries (Supplementary Figures 6A,C). At the genus level, the relative abundances of *Candidatus Nitrososphaera* and *Candidatus Nitrocosmicus* was significantly higher in the nonrhizosphere soil and rhizosphere soil samples of Akihime than in those of *F. nilgerrensis* (*p* < 0.05) (Supplementary Figures 6B,D).

## 4 Discussion

In this study, we compared the compositions, structures, differences, and interactions of microbiome in the nonrhizosphere soil, rhizosphere soil, and root endosphere samples of wild and cultivated strawberry species for the first time. This work aimed to provide reference for the study of strawberry microbiome, germplasm innovation, and disease biocontrol.

### 4.1 The microbiome of wild strawberry *F. Nilgerrensis* Is more abundant and diverse

A number of studies have shown that plants can own their unique microbial communities at the species and even cultivar levels (Schweitzer et al., 2008), and even endophytes that remain in plants for a long time are also different (Anguita-Maeso et al., 2020). For example, the diversity of rhizosphere bacteria in wild soybean was higher than that of cultivated soybean (Ma et al., 2019; Pantigoso et al., 2020), and the diversity of root-related bacteria in wild species, *Oryza rufipogon* was higher than that in cultivated species, *Oryza sativa* (Tian et al., 2017). In addition, the results of Sangiorgio et al. (2022) showed that the organs and genotypes of strawberry plants play an important role in determining the taxonomic and functional composition of microbial communities, which is similar to our results. In the present study, we found that the nonrhizosphere soil and rhizosphere soil samples of *F. nilgerrensis* had higher bacterial diversity and richness than those of Akihime (*p* < 0.05). For the fungal communities, we found that the fungal diversity in the *F. nilgerrensis* samples was significantly higher than that in the Akihime samples (*p* < 0.05) (Table 1). The results also showed that the diversities of bacterial and fungal communities in the soil samples were higher than those in the root endosphere samples of both strawberry species (*p* < 0.05) (Supplementary Table S2), which are similar to the findings on *Populus* (Cregger et al., 2018) and *Agave* (Coleman-Derr et al., 2016). At the same time, the diversities of bacteria and fungi in the two kinds of strawberry rhizosphere soils were higher than those in nonrhizosphere soil, indicating that the presence of host plants did affect the distribution of soil microorganisms (Wei et al., 2021). Univariate correlation network analysis showed that the interaction relationships between bacterial and fungal communities in the *F. nilgerrensis* samples were more complex than those in the Akihime samples (Table 2; Supplementary Figures S3, S4). The high microbial diversities and complex interaction relationships improved the stability of bacterial and fungal communities in the *F. nilgerrensis* samples, consequently enhancing their resistance to pathogens and ecological adaptability (Yang et al., 2020; Liu et al., 2021; Su et al., 2022).

### 4.2 Different microbial communities between wild and cultivated strawberries

In our research, the compositions and structures of the two strawberry microbiomes showed similarities at the phylum level but differences at the genus level, particularly evident in the root microbiomes. These differences may have arisen from the long-term evolution and interaction in different environments and might have been influenced by natural and anthropogenic factors.

At the phylum level, the most abundant bacteria in the rhizosphere soil and nonrhizosphere soil samples of *F. nilgerrensis* and Akihime were *Actinobacteriota*, *Proteobacteria*, *Acidobacteria*, *Chloroflexi*, and *Gemmatimonadota*, and the most abundant in the root endosphere samples was *Proteobacteria*. The most abundant fungal phylum in the *F. nilgerrensis* and Akihime samples was *Ascomycota* (Figures 3A,C,E). The above results are similar to those of other studies (Yang et al., 2020; Su et al., 2022). However, our results for the genera with high relative abundances differ from those of other studies, mainly for the beneficial bacteria genera *Streptomyces*, *Acidibacter*, *Novosphingobium*, *Bradyrhizobium*, *Dongia*, *Pseudomonas*, *Bryobacter*, *Gemmatimonas*, and the fungal communities were mainly the potential pathogenic genera *Fusarium*, *Botrytis*, *Cladosporium* and potentially beneficial genera *Chaetomium*, *Mortierella* (Figures 2, 3) (Yang et al., 2020; Su et al., 2022; Yang et al., 2023).

Compared with plants, soil microbes are more sensitive to climate change, leading to variations in microbial community composition in different environments, such as agricultural fields and mountain soils (Pandey and Yarzábal, 2019; Liu et al., 2022). The rhizosphere serves as a crucial area for plant-microbe interactions, where plant genotypes influence the rhizosphere microbiome, and beneficial microbes in the rhizosphere can influence plant growth through direct or indirect mechanisms (Pandey and Yarzábal, 2019). Additionally, anthropogenic factors such as fertilization, pesticide application, and chemical fumigation can alter the composition of soil microbial communities (Xu et al., 2015; De Tender et al., 2016; Liu et al., 2022). In conclusion, we posit that different genotypes, growth environments, and anthropogenic influences are the reasons behind the microbial community disparities in the nonrhizosphere and rhizosphere soils of wild and cultivated strawberry varieties.

For endophytes, plants recruit diverse microbial communities from the surrounding environment and incorporate them into tissues as endophytes; these recruited bacteria and fungi are typically beneficial microorganisms that play crucial roles in regulating plant development (Berg and Raaijmakers, 2018; White et al., 2019; Jonkers et al., 2022). Meanwhile, symbiotic microbes can be lost during the domestication and long-term cultivation of plants, prompting modern plant varieties to potentially lack certain traits necessary to attract beneficial microbes compared with their wild relatives (Bulgarelli et al., 2015; Irizarry and White, 2017; Abdullaeva et al., 2021). In this study, there are significant differences in the composition and relative abundance of endophytic bacterial and fungal communities in *F. nilgerrensis* and Akihime samples. These differences may stem from the distinct habitats of wild species *F. nilgerrensis* and cultivated Akihime, resulting in variations in root microbial recruits by the strawberries. Adaptations in endophytic microbes during artificial domestication and cultivation may also contribute to these differences. Some bacterial taxa, such as *Pseudomonas* and *Pantoea*, and other potentially beneficial microorganisms are shared between the root endosphere samples of *F. nilgerrensis* and Akihime, illustrating that strawberries can recruit beneficial bacteria and may retain beneficial bacteria during long-term artificial crossbreeding and domestication, which was similar to the results of studies in cereals (Abdullaeva et al., 2021).

### 4.3 Differences in potential pathogens and beneficial bacteria between wild and cultivated strawberries

It was reported that the relative abundances of plant pathogens in cultivated pearl millet were higher than those in wild pearl millet in semi-arid and semi-humid areas (Mofini et al., 2022). A similar phenomenon was observed in strawberries. The relative abundances of potential fungal pathogens including *Fusarium* (Frąc et al., 2018; Gwinn et al., 2022)*, Botrytis* (Drobek et al., 2021; Pandit et al., 2022)*, Rhizoctonia* (Frąc et al., 2018; Pandit et al., 2022)*, Dactylonectria* (Su et al., 2022)*, Cadophora* (Travadon et al., 2015), *Neopestalotiopsis* (Darapanit et al., 2021), and *Colletotrichum* (Drobek et al., 2021; Sangiorgio et al., 2022) were significantly higher in the rhizosphere soil and root endosphere samples of Akihime than those of *F. nilgerrensis* (Figure 5). At the same time, a variety of fungal pathogen taxa were detected in the samples of Akihime with higher relative abundances than *F. nilgerrensis*, especially in the root endosphere samples. These taxa include *Fusarium incarnatum* (which can cause strawberry wilt, root rot and fruit rot) (Ayoubi and Soleimani, 2016), *Rhizoctonia fragariae* (which can cause strawberry root rot) (Erper et al., 2021; Su et al., 2022), *Dactylonectria pauciseptata*, and *Dactylonectria torresensis* (which is associated with black root rot of strawberry) (Chen et al., 2021) (Figure 6). In addition, the potential bacterial pathogens *Erwinia* and *Xanthomonas* (Expert et al., 2004; Timilsina et al., 2020) were also detected in the root endosphere samples of Akihime with higher relative abundances than in *F. nilgerrensis* samples. Although many potential pathogens were detected in the Akihime samples, no evident disease symptoms were observed during the sampling, which is similar to the findings on *Fragaria × ananassa* (Sangiorgio et al., 2022). In this research, we found that the relative abundances of potential biocontrol bacteria such as *Streptomyces, Pseudomonas,* and *flavobacterium* (Su et al., 2022) and potential biocontrol fungi such as *Chaetomium* (Moya et al., 2020) in the rhizosphere soil samples of Akihime were significantly higher than those in the *F. nilgerrensis* samples. On this basis, we speculated that the abundant antagonistic bacteria and fungi in the rhizosphere soil are the reasons why Akihime does not show evident disease symptoms, and the organic fertilizer applied in the field may have been the source of the antagonistic bacteria and fungi (De Tender et al., 2016; Liu et al., 2021; Li et al., 2022a). Although Akihime does not show evident disease symptoms, the risk of underlying diseases remains. Hence, farmers should pay attention to field management and prevent diseases in advance.

It has been hypothesized that modern varieties have a decreased ability to coexist with microbes in response to the highly fertile soil conditions used in the artificial selection of plants (Wissuwa et al., 2009). Similarly, we found that the relative abundances of beneficial bacteria such as *Bradyrhizobium* and *Anaeromyxobacter* associated with nitrogen fixation and ammonization (Zhang et al., 2019; Zhu et al., 2023) were significantly higher in the *F. nilgerrensis* samples than in the Akihime samples (*p* < 0.05). In addition, we found that the genus, *Conexibacter*, which can enhance the resistance of rhizosphere microregions to ecological toxicity and improve microbial community stability in heavy metal-contaminated soils (Chen et al., 2022), had significantly higher relative abundance in the *F. nilgerrensis* soil samples than in the Akihime samples (*p* < 0.05). This situation might be beneficial for *F. nilgerrensis* to adapt to the wild environment. On the contrary, Akihime may have lost the corresponding ability in the process of artificial cultivation. However, researchers have found that even after centuries of domestication and complex hybridization, cultivated strawberry plants still closely interact with 16 genera of nitrogen-fixation bacteria (Sangiorgio et al., 2022). For the nonrhizosphere soil and rhizosphere soil samples, the relative abundances of ammonia oxidizing archaea *Candidatus Nitrososphaera* and *Candidatus Nitrocosmicus* in Akihime was significantly higher than those in *F. nilgerrensis*. These archaea may play important roles in the soil nitrogen cycle of cultivated strawberry (Wu et al., 2021; Kraft et al., 2022; Sun et al., 2022; Liu et al., 2023).

It is well known that the healthy growth of plant roots is critical for nutrient uptake, and Var*iovorax* can reverse root growth inhibition and regulate root growth (Finkel et al., 2020). We found that the relative abundance of *Variovorax* in the root endosphere samples of Akihime was significantly higher than that of *F. nilgerrensis* (*p* < 0.05). This situation is conducive to root growth and nutrient absorption during the cultivation of Akihime. The use of a large number of chemical pesticides, insecticides, fungicides, bactericides, and soil fumigants has a negative impact on soil, water resources, air, and human health (Deng et al., 2019). It is reported that *Blastococcus*, *Solirubrobacter*, *Gemmatimonas*, among others, have the ability to degrade pesticides (Du et al., 2022), while Nocardioides can withstand various low-nutrient conditions and concurrently degrade pollutants (Ma et al., 2023). We observed that the relative abundances of *Blastococcus, Nocardioides, Solirubrobacter*, and *Gemmatimonas* in the rhizosphere soil samples of Akihime were significantly higher than those of *F. nilgerrensis* (*p* < 0.05). This trend may be attributed to the response of soil microbes to harmful chemical residues such as pesticides during the cultivation of Akihime. In this regard, some viewpoints suggest that cultivated plants may recruit microorganisms specialized in carrying out beneficial functions under artificial cultivation conditions, and the ability to interact with these beneficial microbes might be a trait acquired during the domestication and adaptation to field environments (Sangiorgio et al., 2022). This perspective elucidates the presence of beneficial microorganisms in the Akihime samples that are absent in the *F. nilgerrensis* samples to some extent.

### 4.4 Strawberry root endophytic microbiome is an important source of biocontrol and beneficial microorganisms

The biological control of bacteria, fungi, and related beneficial microorganisms has been widely used in crop cultivation (Tariq et al., 2020; Collinge et al., 2022; Sangiorgio et al., 2022). *Ampelomyces quisqualis, Bacillus subtilis* and *Trichoderma harzianum* have been applied to control strawberry diseases. However, they have not achieved the effect of replacing chemical pesticides (Husaini and Neri, 2016), which may be due to the fact that the microorganisms used are not obtained from strawberry microflora. Studies have shown that biocontrol bacteria isolated directly from plant microorganisms have higher efficacy than other “non-self” sources (Haney et al., 2015; Sangiorgio et al., 2022). We found abundant potential beneficial bacteria including *Pseudomonas, Pantoea, Sphingomonas, Streptomyces, Variovorax, Blastococcus,* and *Enterobacter* in the root endosphere samples of the two strawberry species, especially in the root endosphere samples of *F. nilgerrensis*. This finding indicates that strawberry plants can recruit beneficial bacteria from soil and form symbionts to enhance their environmental adaptability and resistance to pathogens. Therefore, strawberry root endophytic microorganisms are important sources of beneficial bacteria, and researchers can try to isolate related beneficial bacteria from the roots of healthy *F. nilgerrensis* for the prevention and control of strawberry diseases.

## 5 Conclusion

In this study, amplicon-based next-generation sequencing was used to comparatively study the microbiomes of wild species *F. nilgerrensis* and cultivated variety Akihime. We found that the richness of bacteria and fungi in the samples of *F. nilgerrensis* were higher than that in the Akihime samples. In terms of diversity, the bacterial and fungal communities in the nonrhizosphere soil and rhizosphere soil of *F. nilgerrensis* were more diverse than those in the Akihime samples. Additionally, the relative abundances of microbial groups associated with nitrogen fixation and ammonization, and those related to ecological toxicity resistance and microbial community stability were significantly higher in the nonrhizosphere and rhizosphere soils of *F. nilgerrensis* compared with those in the Akihime samples. Moreover, we found that many potential pathogen genera and beneficial genera were more abundant in the Akihime samples than in the *F. nilgerrensis* samples. Similarly, the relative abundances of ammonia-oxidizing archaea and beneficial bacteria related to pesticide degradation and root growth regulation were higher in the nonrhizosphere and rhizosphere soils of Akihime than those of *F. nilgerrensis*. These microbial groups may help Akihime adapt to farmland environments. It is worth noting that the root endophytic microbiome of *F. nilgerrensis* was mainly composed of potential biocontrol and beneficial bacteria genera, which are an important source of isolating biocontrol and beneficial microbes. Our results provide theoretical guidance and data support for the biological control of strawberry diseases and the isolation of growth-promoting and disease-resistant strains.

## Data availability statement

The datasets presented in this study can be found in online repositories. The names of the repository/repositories and accession number(s) can be found at: https://www.ncbi.nlm.nih.gov/, PRJNA1070056.

## Author contributions

ZW: Conceptualization, Data curation, Formal analysis, Investigation, Methodology, Project administration, Software, Visualization, Writing – original draft, Writing – review & editing. QD: Conceptualization, Data curation, Formal analysis, Investigation, Project administration, Validation, Visualization, Writing – original draft, Writing – review & editing. DS: Conceptualization, Investigation, Methodology, Software, Validation, Writing – review & editing. ZZ: Investigation, Methodology, Writing – original draft. YT: Investigation, Writing – original draft. JT: Funding acquisition, Supervision, Writing – review & editing. SY: Investigation, Methodology, Writing – review & editing. CY: Investigation, Methodology, Writing – original draft. JY: Conceptualization, Funding acquisition, Investigation, Writing – review & editing. XC: Funding acquisition, Investigation, Project administration, Resources, Supervision, Validation, Writing – review & editing.

## Supplementary material

Supplementary Figure 1 The dilution curve of different samples (*n* = 3) based on Shannon index of bacterial **(A,B,C)** and fungal **(D,E,F)** communities.

Supplementary Figure 2 PLS-DA analysis of different samples based on bacterial **(A,B,C)** and fungal **(D,E,F)** OTUs.

Supplementary Figure 3 Single factor correlation network analysis of bacterial communities in the samples of *F.nilgerrensis* **(A,B,C)** and Akihime **(D,E,F)**.

Supplementary Figure 4 Single factor correlation network analysis of fungal communities in the samples of *F.nilgerrensis* **(A,B,C)** and Akihime **(D,E,F)**.

Supplementary Figure 5 Relative abundances of archaeal community compositions at the phylum **(A,C,E)** and genus **(B,D,F)**levels are determined in different samples (*n* =  3). The genus and phylum with relative abundance <1% is combined into“others”.

Supplementary Figure 6 Analysis of differences in the relative abundance of archaeal communities in different samples (*n* = 3) at phylum **(A,C)** and genus **(B,D)**levels.
