# Supplementary material for: Comparative analysis of the microbiomes of strawberry wild species Fragaria nilgerrensis and cultivated variety Akihime using amplicon-based next-generation sequencing
Source: Front Microbiol. 2024 May 30;15:1377782. doi: 10.3389/fmicb.2024.1377782 (PMC11169695; doi:10.3389/fmicb.2024.1377782)
Supplement: Supplementary Figure 1 — The dilution curve of different samples (n = 3) based on Shannon index of bacterial (A,B,C) and fungal (D,E,F) communities. [file Data_Sheet_1.zip › Figure legends.docx]

Supplementary Figure 1: The dilution curve of different samples (n=3) based on Shannon index of bacterial (A,B,C) and fungal (D,E,F) communities.

Supplementary Figure 2: PLS-DA analysis of different samples based on bacterial(A,B,C) and fungal (D,E,F) OUTs.

Supplementary Figure 3: Single factor correlation network analysis of bacterial communities in the samples of *F.nilgerrensis* (A,B,C) and Akihime (D,E,F.).

Supplementary Figure 4: Single factor correlation network analysis of fungal communities in the samples of *F.nilgerrensis* (A,B,C) and Akihime (D,E,F.).

Supplementary Figure 5: Relative abundances of archaeal community compositions at the phylum (A,C,E) and genus (B,D,F) levels are determined in different samples (n = 3). The genus and phylum with relative abundance <1% is combined into“others”.

Supplementary Figure 6: Analysis of differences in the relative abundance of archaeal communities in different samples (n=3) at phylum (A,C) and genus (B,D) levels.
