# Supplementary figures and images for: Comparative analysis of the microbiomes of strawberry wild species Fragaria nilgerrensis and cultivated variety Akihime using amplicon-based next-generation sequencing
Source: Front Microbiol. 2024 May 30;15:1377782. doi: 10.3389/fmicb.2024.1377782 (PMC11169695; doi:10.3389/fmicb.2024.1377782)

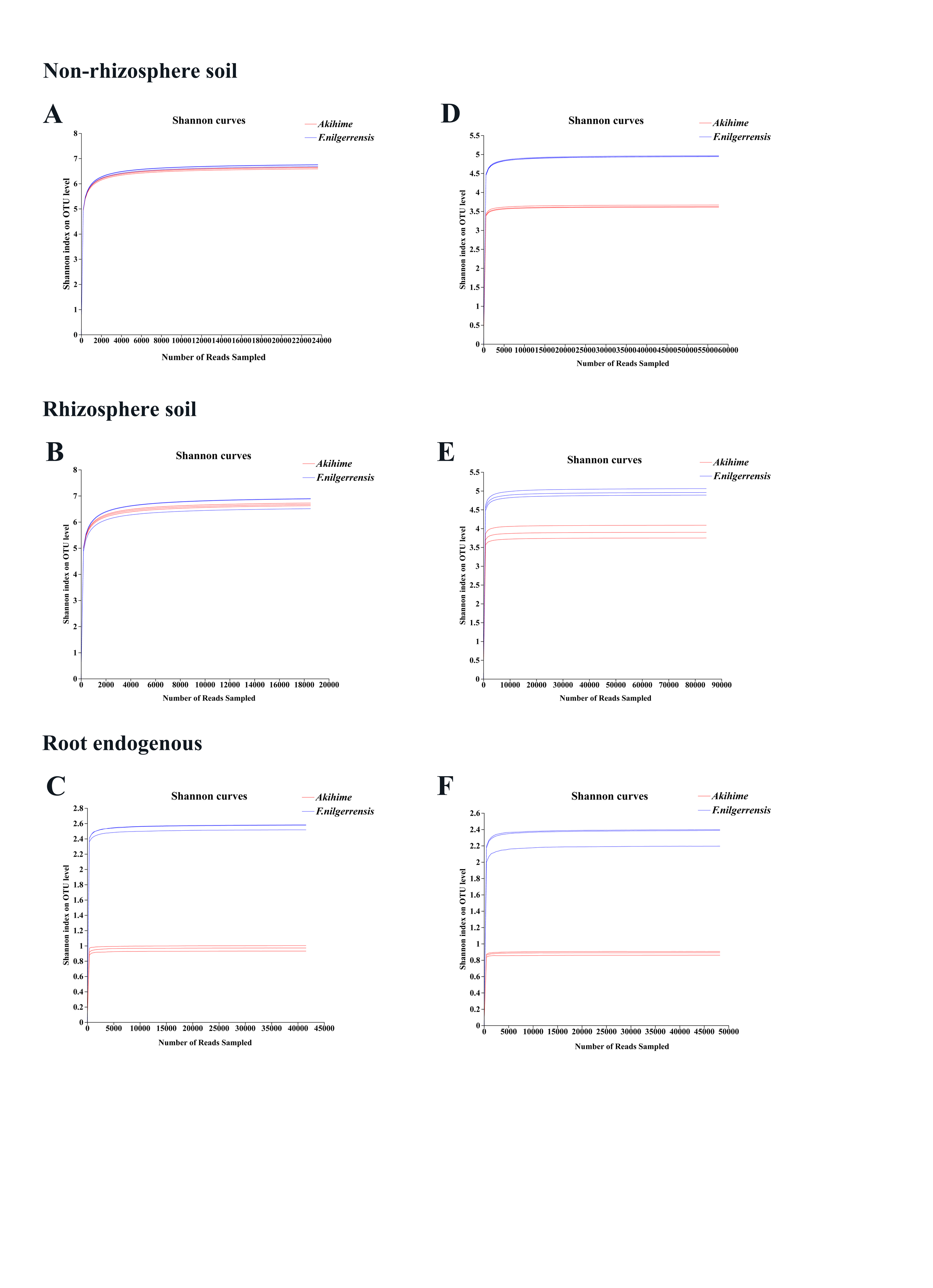

Supplement: Supplementary Figure 1 — The dilution curve of different samples (n = 3) based on Shannon index of bacterial (A,B,C) and fungal (D,E,F) communities. [file Data_Sheet_1.zip › Supplementary Figure 1 The dilution curve of different samples (n=3) based on Shannon index of bacterial (A,B,C) and fungal (D,E,F) communities.jpg]

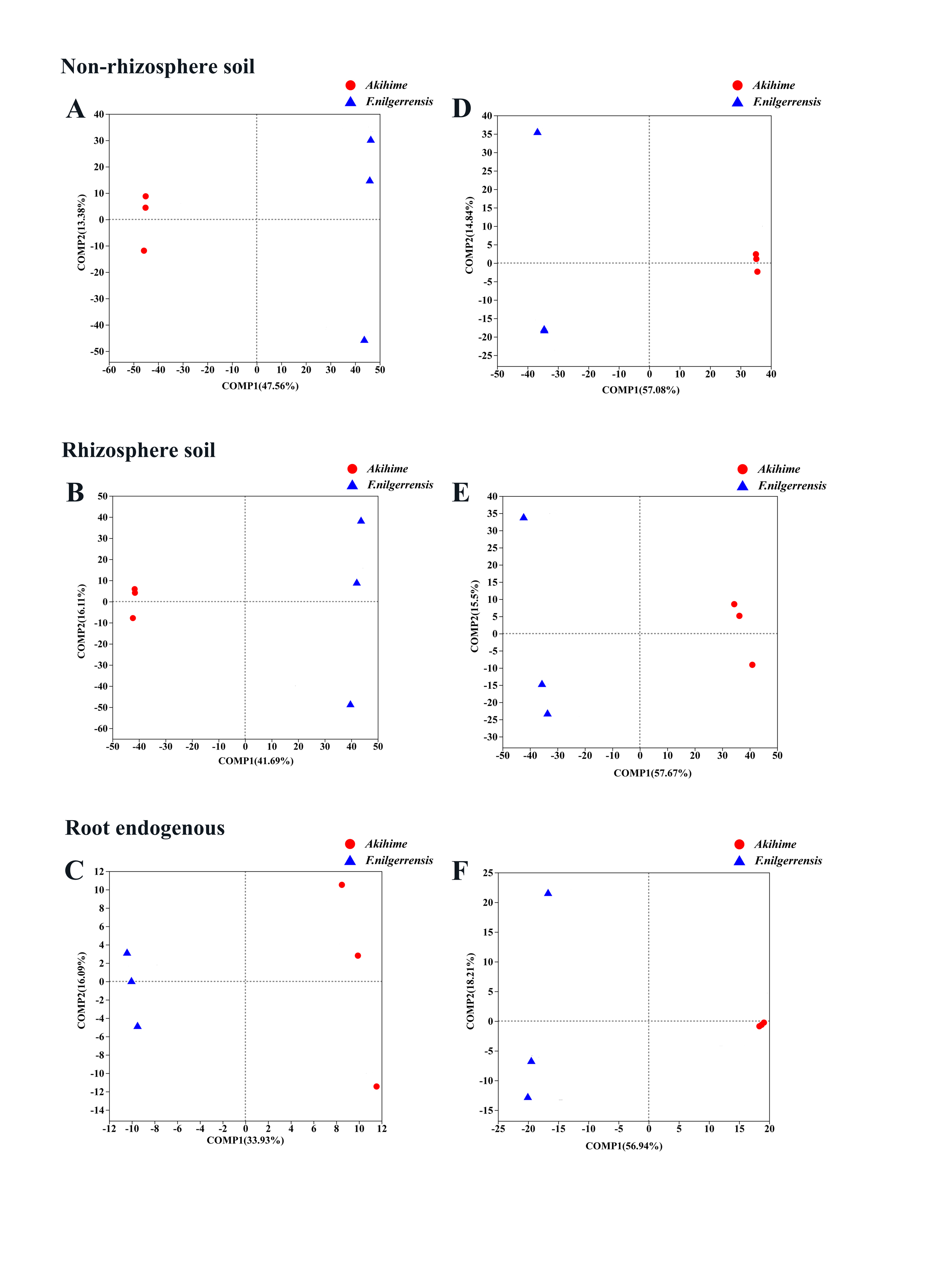

Supplement: Supplementary Figure 1 — The dilution curve of different samples (n = 3) based on Shannon index of bacterial (A,B,C) and fungal (D,E,F) communities. [file Data_Sheet_1.zip › Supplementary Figure 2 PLS-DA analysis of different samples based on bacterial(A,B,C) and fungal (D,E,F) OUTs.jpg]

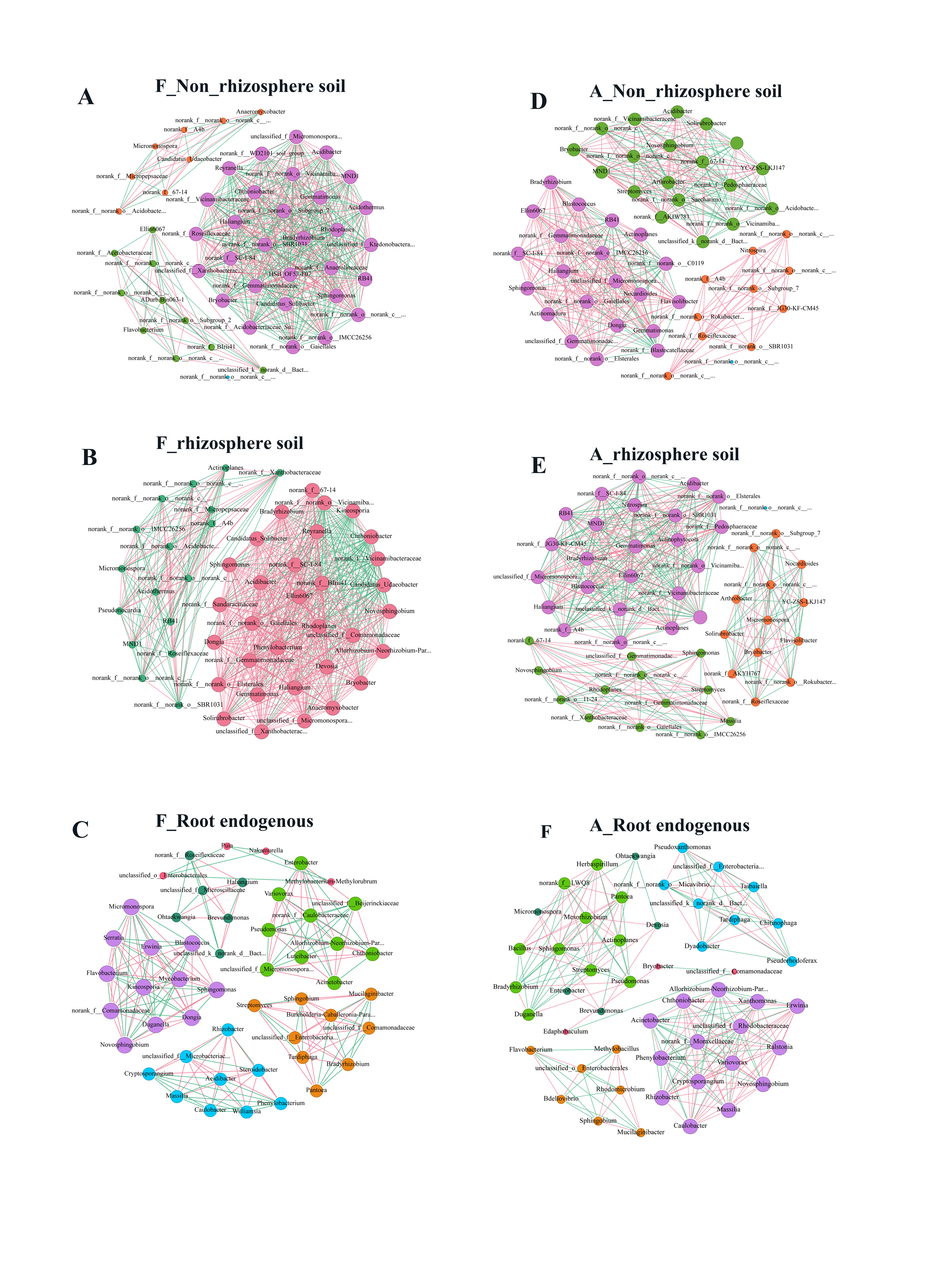

Supplement: Supplementary Figure 1 — The dilution curve of different samples (n = 3) based on Shannon index of bacterial (A,B,C) and fungal (D,E,F) communities. [file Data_Sheet_1.zip › Supplementary Figure 3 Single factor correlation network analysis of bacterial communities in the samples of F.nilgerrensis (A,B,C) and Akihime (D,E,F.).jpg]

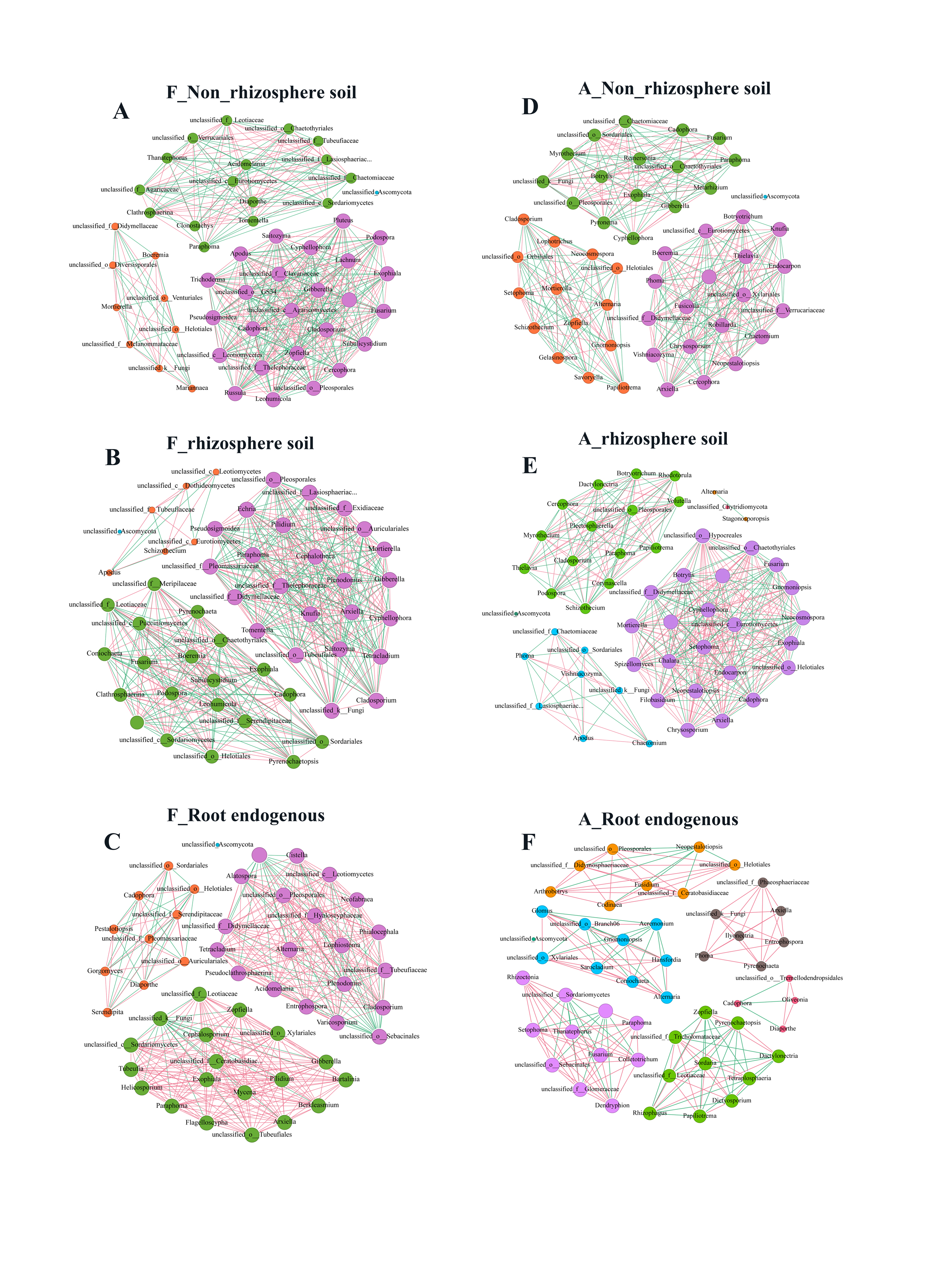

Supplement: Supplementary Figure 1 — The dilution curve of different samples (n = 3) based on Shannon index of bacterial (A,B,C) and fungal (D,E,F) communities. [file Data_Sheet_1.zip › Supplementary Figure 4 Single factor correlation network analysis of fungal communities in the samples of F.nilgerrensis (A,B,C) and Akihime (D,E,F.).jpg]

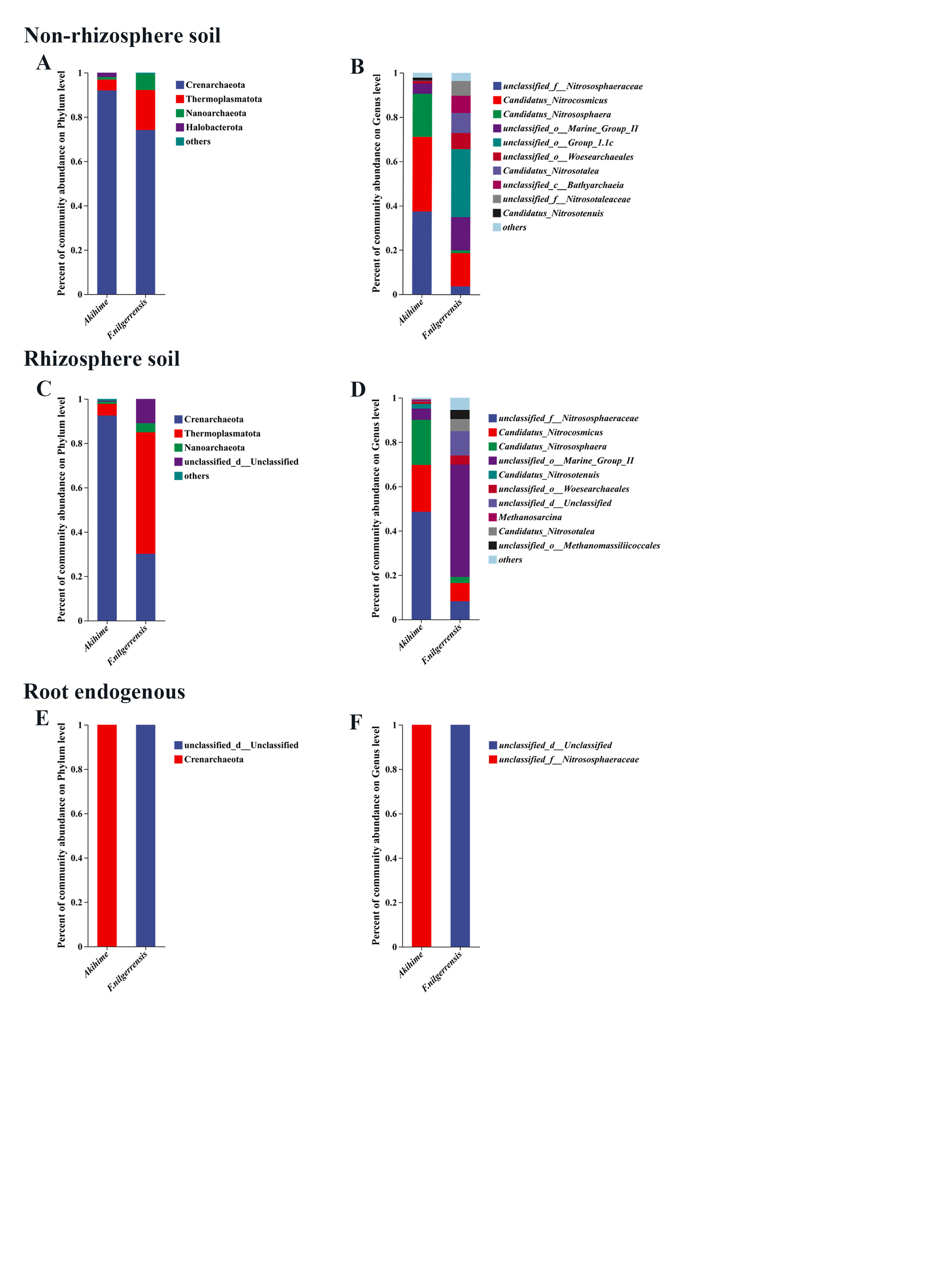

Supplement: Supplementary Figure 1 — The dilution curve of different samples (n = 3) based on Shannon index of bacterial (A,B,C) and fungal (D,E,F) communities. [file Data_Sheet_1.zip › Supplementary Figure 5 Relative abundances of archaeal community compositions at the phylum (A,C,E) and genus (B,D,F) levels.jpg]

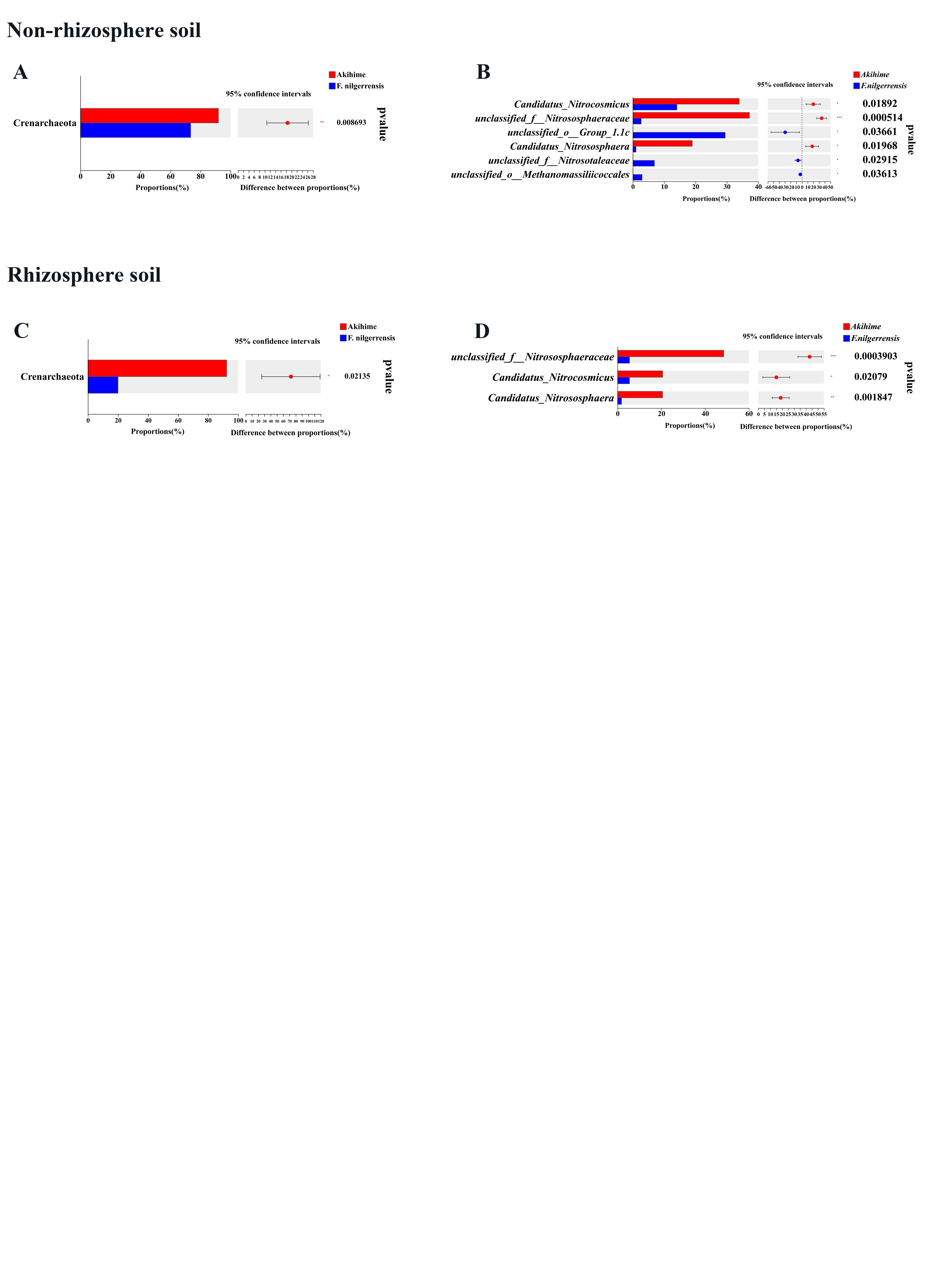

Supplement: Supplementary Figure 1 — The dilution curve of different samples (n = 3) based on Shannon index of bacterial (A,B,C) and fungal (D,E,F) communities. [file Data_Sheet_1.zip › Supplementary Figure 6 Analysis of differences in the relative abundance of archaeal communities in different samples (n=3) at phylum (A,C) and genus (B,D) levels.jpg]
