# Supplementary material for: Comparative analysis of the microbiomes of strawberry wild species Fragaria nilgerrensis and cultivated variety Akihime using amplicon-based next-generation sequencing
Source: Front Microbiol. 2024 May 30;15:1377782. doi: 10.3389/fmicb.2024.1377782 (PMC11169695; doi:10.3389/fmicb.2024.1377782)
Supplement: Supplementary file 2 [file Table_1.docx]

Supplementary Table 1 Overview of high throughput sequencing

| Amplification region | Sample names | clean reads number | OUT number | coverage |
| --- | --- | --- | --- | --- |
| 16S | A_Non_rhizosphere soil | 41445.33±9889.0757a | 2539.67±22.8108a | 0.9635±0.00066 |
|  | F_Non_rhizosphere soil | 41959.33±6580.4551a | 2660.67±56.0833b | 0.9629±0.00226 |
|  | A_rhizosphere soil | 36808.00±4718.9657a | 2327.33±27.5379a | 0.9556±0.00032 |
|  | F_rhizosphere soil | 36697.67±534.3261a | 2567.67±223.2405a | 0.9503±0.00366 |
|  | A_ Root endogenous | 43410.67±1577.5996a | 111.33±5.6862a | 0.9745±0.00518 |
|  | F_ Root endogenous | 68735.00±1487.4082b | 124.00±3.4641b | 0.9613±0.00440 |
| ITS2 | A_Non_rhizosphere soil | 94905.33±3283.4571a | 662.33±9.4516a | 0.9970±0.00013 |
|  | F_Non_rhizosphere soil | 71769.33±10674.6590a | 1534.33±21.3620b | 0.9928±0.00030 |
|  | A_rhizosphere soil | 104427.00±8331.2916a | 736.67±51.3939a | 0.9984±0.00021 |
|  | F_rhizosphere soil | 101541.33±13141.2264a | 1752.33±64.0495b | 0.9945±0.00013 |
|  | A_ Root endogenous | 56066.00±6371.3015a | 63.00±4.3590a | 0.9996±0.00011 |
|  | F_ Root endogenous t | 102927.67±6350.8930b | 510.67±12.7017b | 0.9973±0.00012 |

A_, Akihime sample; F_, *Fragaria nilgerrensis* (M±SD, n = 3). T values are based on independent sample t-tests. Significance is indicated by different letters.

Supplementary Table 2 Comparison of α diversity indices of bacterial and fungal communities in different samples of the same species

| **Amplification region** | **Sample names** | **community diversity** | |  | **community richness** | | |
| --- | --- | --- | --- | --- | --- | --- | --- |
|  |  | **Shannon** | **Simpson** |  | **Ace** | **Chao** | **Sobs** |
| **16S** | **A_Non_rhizosphere soil** | **6.623±0.0357a** | **0.0035±0.00014a** |  | **3472.601±53.308a** | **3436.881±85.732a** | **2539.667±22.811a** |
|  | **A_rhizosphere soil** | **6.671±0.0514a** | **0.0029±0.00027a** |  | **3234.718±6.834a** | **3197.135±14.847b** | **2327.333±27.538b** |
|  | **A_ Root endogenous** | **2.987±0.0846b** | **0.0906±0.00703b** |  | **248.538±92.245b** | **194.893±56.144c** | **111.3333±5.687c** |
|  | **F_Non_rhizosphere soil** | **6.723±0.0397a** | **0.0035±0.00034a** |  | **3569.328±140.850a** | **3566.978±237.504a** | **2660.667±56.083a** |
|  | **F_rhizosphere soil** | **6.760±0.2197a** | **0.0039±0.00150a** |  | **3572.441±277.268a** | **3550.256±253.285a** | **2567.667±223.241a** |
|  | **F_ Root endogenous** | **2.414±0.0256b** | **0.1946±0.00222b** |  | **488.949±166.227b** | **320.794±91.527b** | **124.000±3.464b** |
| **ITS2** | **A_Non_rhizosphere soil** | **3.630±0.0287a** | **0.0950±0.00335a** |  | **826.550±21.133a** | **827.842±22.900a** | **662.333±9.452a** |
|  | **A_rhizosphere soil** | **3.909±0.1699b** | **0.0479±0.00698b** |  | **834.834±51.576a** | **829.853±47.579a** | **736.667±51.394b** |
|  | **A_ Root endogenous** | **0.884±0.0232c** | **0.5577±0.00616c** |  | **107.000±29.135b** | **91.833±25.342b** | **63.000±4.359c** |
|  | **F_Non_rhizosphere soil** | **4.949±0.0131a** | **0.0352±0.00073a** |  | **1925.258±28.521a** | **1915.721±53.372a** | **1534.333±21.362a** |
|  | **F_rhizosphere soil** | **4.967±0.0868a** | **0.0312±0.00344a** |  | **2187.663±56.286b** | **2194.218±39.893b** | **1752.333±64.049b** |
|  | **F_ Root endogenous** | **2.326±0.1143b** | **0.3867±0.02889b** |  | **622.714±18.071c** | **617.057±27.908c** | **510.667±12.702c** |

A_, Akihime sample; F_, *Fragaria nilgerrensis* (M±SD, n = 3). T values are based on independent sample t-tests. Significance is indicated by different letters.
